# Using abandoned unripe grape resources to solve the low-acid problem in the northwest wine region of China

**DOI:** 10.1016/j.fochx.2023.100976

**Published:** 2023-10-31

**Authors:** Mengyuan Wei, Yue Tian, Kekun Zhang, Lei Wang, Qian Ge, Tingting Ma, Yulin Fang, Xiangyu Sun

**Affiliations:** a College of Enology, Shaanxi Provincial Key Laboratory of Viti-Viniculture, Viti-viniculture Engineering Technology Center of State Forestry and Grassland Administration, Shaanxi Engineering Research Center for Viti-Viniculture, Heyang Viti-viniculture Station, Ningxia Eastern Foot of Helan Mountain Wine Station, Northwest A&F University, Yangling 712100, China; b College of Food Science and Engineering, Northwest A&F University, Yangling 712100, China; c Yinchuan Institute of Industrial Technology, Yinchuan 750002, China; d Quality Standards and Testing Institute of Agricultural Technology, Ningxia Academy of Agricultural Sciences, Yinchuan 750002, China

**Keywords:** Abandoned unripe grape, Low acid problem, Northwest wine region of China, Antioxidant capacity, Sensory quality

## Abstract

Low acid is the main defect in the northwest wine region of China in recent years. The fermentation of unripe grape (UG) and wine grapes with low acid contents was carried out. Compared with control group (CK), the addition of UG addressed the core flaw that low acid grape bring to wine firstly, it significantly increased titratable acid, tartaric acid and malic acid while significantly decreasing alcohol and volatile acids in wine. Secondly, UG significantly improved wine color, the color parameters a*, b*, C* and L* were significantly increased to different degrees. At the same time, the addition of UG significantly improves other qualities of wine, including the phenolic substances and antioxidant capacity of wine. In addition, adding UGJ2% significantly improved the sensory quality, and pleasant volatile substances such as phenethyl alcohol, ethyl hexanoate, ethyl butyrate and isoamyl acetate were significantly increased, giving the wine more prominent floral and fruity aromas.

## Highlights

• Adding unripe grape (UG) solved the core flaw of low-acid wine materials in northwest China region.
• UG increased titratable acid, tartaric acid and malic acid, decreased volatile acids.
• UG significantly promoted bathochromic shifts, yellowing and shallowing in wine.
• UG significantly increased the phenolic substances and antioxidant capacity of wine.
• By giving more prominent floral & fruity aromas, UGJ2% improved wine sensory quality.

## Graphical abstract

**Figure:**
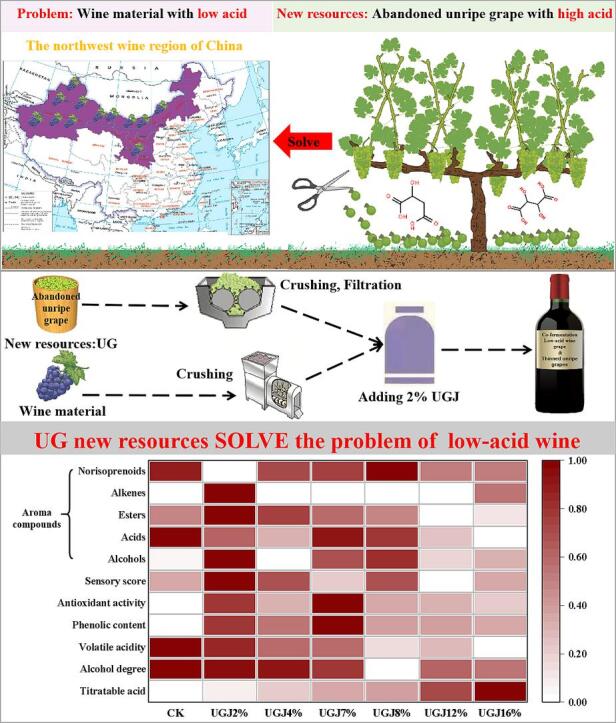

## 1 Introduction

The northwest wine region of China is the main and advantageous producing area of wine industry in China. During grape growth, the permeability of the cell membrane gradually increases so that the acid stored in the cell vacuole is respirated and converted from acid to sugar (Jediyi et al., 2019). As temperature rises and light increases, the accumulation of sugars in wine grapes accelerates, organic acid content decreased, resulting in wine having the disadvantages of high sugar and low acid, color aging rapidly, low in roundness, unbalanced taste and freshness (Gutiérrez et al., 2021). At present, the imbalance of sugar-acid in wine grapes caused by global warming has become a bottleneck problem limiting the development in the northwest wine region of China.

In response to the great challenges posed by the natural environment to the wine industry, previous research has been conducted from several perspectives. First of all, during the cultivation of wine grapes, the acidity of wine grapes could be increased by altering the partial defoliation in shaded canopies (Coniberti et al., 2012), using elicitors (Gutiérrez et al., 2021), reducing irrigation (Yang et al., 2019) and so on. However, above cultivation practices could not achieve directional control and it is uncertain whether it negatively affected other quality indicators. Secondly, during the overall fermentation processes, tartaric acid or ion exchange resins were added to improve the sugar-acid balance of wines during the winemaking process (Ponce, Mirabal-Gallardo, Versari, and Laurie, 2018). Although the addition of chemical reagents can alleviate the defect of low acidity of wine to a certain extent, it also brings the problem of homogeneity of wine. In addition, researchers have screened acid-producing *Saccharomyces* or non-*Saccharomyces* to solve the problem of low acid content of wine grape (Kemsawasd et al., 2015, Vaquero et al., 2021), but acid production efficiency is a major problem restricting the application of this method.

In addition to exogenously added chemical reagents, natural raw materials that could be added into wine were also constantly sought, such as Gordillo et al. (2014) added white grape pomace to red grape mash, which significantly improved the content of phenolic substances and the stability of anthocyanins in wine. For wines with defects of high sugar and low acid, abandoned unripe grape (UG), which has been widely studied recently, may be one of the best natural exogenous additives to improve the quality of low acid wines. Previous studies showed that about 14436.16 kt worldwide of unripe grape was abandoned in orchards every year (Wei et al., 2022), the abandonment of UG is not only a great waste of agricultural resources but also places great pressure on the environment. In fact, UG are rich in organic acids, phenolic substances and other bioactive components (Wei et al., 2021), it can replace exogenous chemical reagents such as tartaric acid, tannin and antioxidant in the winemaking process to supplement the lack of various homologous components in low-acid wine grapes, which has a positive effect on the production of low-acid wines in terms of quality, efficiency and cost savings. Studies have shown that the fermentation of UG with wine grapes, which have high sugar and low acid contents, could increase the acidity and reduce the alcohol content of wine (Piccardo et al., 2019), thus effectively alleviating the overripening problem of wine grapes caused by climate change. Therefore, UG is a potential resource to improve low acid wines with high sugar and low acid, color aging rapidly, low in roundness, unbalanced taste and freshness.

Current studies have focused on the effects of UG on individual factors such as acidity, alcohol or color, but wine is a complex colloidal system and the effects of the addition of UG mixture on overall wine quality remain unknown. In addition, the proportion of UG added in wine grapes is still key issues that need to be further explored. Therefore, UG was mixed for fermentation with wine grapes in this experiment, and the effects of different additions of UG on the overall quality of wine were explored from the aspects of physicochemical indicators, functional characteristics and sensory quality to lay a further theoretical foundation and technical reference for its industrial application. While improving the raw material for winemaking, which has a high sugar content and low acidity, the potential agricultural resources of thinned unripe grapes have also been effectively utilized to promote the green and sustainable development of the grape and wine industry.

## 2 Materials and methods

### 2.1 Grape materials and winemaking

The research was conducted using *Vitis labrusca* × *vinifera* cv. Kyoho and *Vitis vinifera* L. cv. Cabernet Sauvignon. During the thinning period (15 days after flowering), Kyoho was collected in Chongzuo, China, in 2020 (21°36′ N, 106°33′ E). Thinned unripe grapes were pulped with a homogenizer (H-AE-DNBI1, Hurom, Korea). After filtration using three layers of gauze, the unripe grape juice (UGJ) was stored at −40 ℃ for later.

Cabernet Sauvignon came from Chateau Lilan at the eastern base of Mountain Helan, Yinchuan, China (38°28′ N, 105°97′ E), in 2020. When the grapes are commercially ripe (106 days after flowering), 30 kg Cabernet Sauvignon was collected for vinification per treatment. The frozen UGJ was removed and dissolved, and was fermented with Cabernet Sauvignon together.

The vinification method was performed according to Cheng et al. (2020a) with minor modifications. There were 7 groups, all of which adopted the same winemaking process. A total of 30 L must was added to 7 stainless steel fermenting tanks with temperature control devices, followed by the addition of UGJ to each group at 0 % (CK), 2 % (UGJ2%), 4 % (UGJ4%), 7 % (UGJ7%), 8 % (UGJ8%), 12 % (UGJ12%) and 16 % (UGJ16%) of the volume of must. After 24 h of maceration, 200 mg/L commercial yeast was added. The must was monitored and punch down two times a day, and its specific gravity was recorded to ensure normal fermentation progress, and its temperature was recorded and controlled at a temperature of 25–28 ℃. When residual sugar in the must was below 2 g/L, alcoholic fermentation was finished.

During the overall fermentation processes, a total of 8 times were sampled, and the sampling periods were pre-fermentation (PFM), early stage of fermentation (3 days after the start of alcoholic fermentation, 1-FM), later stage of fermentation (7 days after the start of alcohol fermentation, 2-FM), end of fermentation (15 days after the start of alcohol fermentation, E-FM), time of drawing off (DO), time of bottling (BW), after aging for 6 months (AG-1) and after aging for 12 months (AG-2); the samples were then stored at −40 °C. Organic acids, phenolic components and color characteristics were dynamically monitored during the whole process of winemaking. The physicochemical properties of wine were measured in the BW period, and the antioxidant capacity, volatile components and sensory evaluation of wine were measured in the AG-2 period.

### 2.2 Analysis of physicochemical indices in wines

Total soluble solid (TSS), pH value, reducing sugars and titratable acid (TA) were measured using the methods of OIV (International Organization of Vine and Wine) (OIV, 2017). Organic acids (oxalic acid, tartaric acid, quinic acid, malic acid, shikimic acid, lactic acid, acetic acid, citric acid, fumaric acid, succinic acid, and propionic acid) were determined by HPLC strictly following the methods of Wei et al. (2022). Fermentation products such as alcohol, volatile acid, glycerol, glucose and fructose were determined by HPLC strictly following the methods of Moreira, Mendes, Hogg, and Vasconcelos (2005). Standards for monomer organic acids and fermentation products were purchased from Sigma–Aldrich (MO, USA).

### 2.3 Colorimetric analysis

A W100 colorimeter analyzer (Haineng Instrument Co., Ltd., China) was used to analyze the color parameters of wine in reflection mode. On the CIELAB scale, the lightness (L*), green/red component (a*), yellow/blue component (b*) and chroma (C*) of the samples were recorded.

### 2.4 Analysis of phenolic substances and antioxidant activities

The total polyphenol content (TPC), flavonoid content (TFC), flavan-3-ol content (TFO) and anthocyanin content (TAC) were measured by the Folin-Ciocalteu colorimetric method, aluminum chloride colorimetric assay, *p*-(dimethylamino) cinnamaldehyde (*p*-DMACA)-HCl assay and pH differential method, respectively. They were measured according to the literature of Meng et al. (2012). The results were expressed as milligrams of gallic acid equivalents per L (mg GAE/L), milligrams of rutin equivalents per L (mg RTE/L), milligrams of catechin equivalents per L (mg CTE/L), and milligrams of malvidin-3-*O*-glucoside equivalents per L (mg Mv/L). Gallic acid, rutin, catechin and so on were purchased from Yuanye Bio-Technology Co., Ltd. (Shanghai, China).

The monomeric anthocyanin was determined by HPLC, and the samples were filtered through a 0.22 μm organic membrane before determination and detected by a Shimadzu LC-20A liquid chromatograph (Kyoto, Japan) with a Synergi Hydro RP C_18_ column (250 mm × 4.6 mm, 4 μm). Mobile phases A and B were formic acid: acetonitrile: water = 2.5: 10: 80 V/V/V and formic acid: acetonitrile: water = 2.5: 50: 40 V/V/V, respectively. The elution gradient was as follows: 0–45 min, 0–35 % B; 45–46 min, 35–100 % B; 46–50 min, 100–100 % B; 50–51 min, 100–0 % B; and 51–55 min, 0–0 % B. The flow rate was 1 mL/min, and the detection wavelength was 520 nm. The relative concentration of each anthocyanin was expressed as the malvidin-3-*O*-glucoside equivalent. Malvidin-3-*O*-glucoside was purchased from Sigma-Aldrich (MO, USA).

Total antioxidant capacity (*T*-AOC) and ABTS radical scavenging capacity were determined using a kit (Solarbio Science & Technology Co., Ltd., Beijing, China). The copper ion reducing capacity (CUPRAC) was determined by the methods of Cheng et al. (2020b). The results of *T*-AOC and CUPRAC were expressed in millimoles of trolox equivalents per L (mmol TEAC/L), and the others were expressed as percentages (%).

### 2.5 Sensory quality analysis

#### 2.5.1 Sensory evaluation

A sensory analysis trial was performed in a purpose-built sensory room in the College of Enology, Northwest A&F University. Wines from seven different treatments were tested by a group of expert laboratory staff experienced in sensory analysis (14 members; 7 males and 7 females from 23 to 45 years old). The comprehensive sensory quality of wine was evaluated according to the detailed evaluation criteria in Table S1.

#### 2.5.2 Volatile organic compounds (VOCs)

A solid-phase microextraction head coupled with gas chromatography–mass spectrometry (HS–SPME–GC–MS) was used to analyze the VOCs in the wine samples. A 5 mL wine sample and 10 μL of internal standard solution (1.0083 g/L, 4-methyl-2-pentanol) were placed in a headspace bottle containing 1 g NaCl. The sample was equilibrated in the vibrator at 250 r/min and 40℃ for 5 min. The aged extracted fibers (50/30 μm, DVB/CAR/PDMS, Supelco, Bellefonte, USA) were inserted into a headspace bottle containing the sample for extraction for 30 min and desorbed at 250℃ for 10 min.

The GC–MS analyses were performed on a GC–MS TQ8050 NX system (Shimadzu, Kyoto, Japan) equipped with a DB-WAX-UI capillary column (60.0 m × 0.25 mm × 0.25 μm, Shimadzu, Kyoto, Japan), and the carrier gas was helium at a flow rate of 1.0 mL/min. Heating procedure: The initial temperature was 40℃ and held for 3 min, and it was then increased to 160℃ at a rate of 4℃/min. Then, the temperature was increased to 230 °C at a rate of 7 °C/min and held for 8 min. The injection volume was 1 μL, the ion source temperature and ion energy were 230 °C and 70 eV, and the mass spectrum scanning range was 33–450 *m*/*z* in full scan mode.

According to the NIST14 mass spectrometry library, the compounds were preliminarily identified based on the *n*-alkane mixture, retention time, mass spectrometry and 70 % matching degree, and the qualitative results of the compounds were further validated with relevant literature reports. For compounds with standards, the external standard quantitative method was used for the quantitative analysis of compounds. For compounds without standards, 4-methyl-2-pentanol was used as an internal standard substance for semiquantitative analysis to obtain the concentration of volatile compounds (μg/L). The odor activity value (OAV) was calculated by the ratio between the concentration of the compound and its sensory threshold (Li et al., 2023, Van Heimert, 2015), as shown in Table S2. When OAV > 1, this compound was considered to be a key aroma compound (Lan et al., 2022).

### 2.6 Statistical analysis

The results were expressed as the mean ± standard deviation of the three measurements. SPSS 20 (IBM, USA) was used for one-way analysis of variance (ANOVA) and Duncan’s multivariate test (*p* < 0.05). GraphPad Prism 8 (GraphPad Software, USA), Origin 9.1 (OriginLab, USA) and TBtools were used for image rendering and data analysis.

## 3 Results and discussion

### 3.1 Effects of UG on acidity and others enological parameters of low acid wine

Studies have shown that wine could achieve the best balance of sugar-acid when the sugar-to-acid ratio of grapes was between 30 and 35 (Schrader, Cochran, Domoto, and Nonnecke, 2020). However, as shown in Table S3, the reducing sugar and TA contents of Cabernet Sauvignon from the northwest wine region of China were 255.50 g/L and 5.10 g/L, respectively, and the sugar-to-acid ratio was as high as 50, so the wine grapes obviously had high sugar and low acid contents. The addition of UGJ significantly improves the core problem of low-acid wine, compared with CK, the addition of UGJ significantly reduced the pH value of the wine, and the TA content of the wine was significantly increased except for the UGJ2% group (*p* < 0.05) (Table S4).

**Fig. 1:**
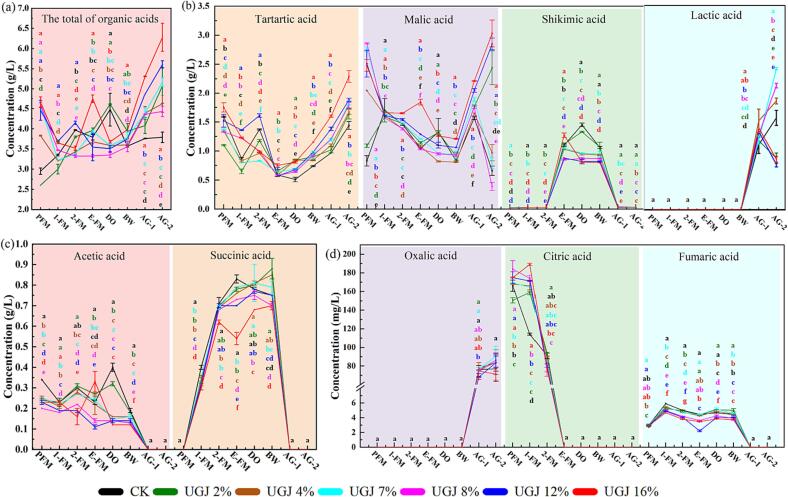
The analysis of organic acids in wines with different UGJ additions during the winemaking period (a) total organic acids; (b) tartaric acid, malic acid, shikimic acid and lactic acid; (c) acetic acid and succinic acid; (d) oxalic acid, citric acid and fumaric acid. Note: The different small letters indicate a significant difference among the different groups of wine (*p* < 0.05).

Organic acids have always been underestimated chemical components in wine;, organic acids also can affect the sugar and acid balance, color characteristics and overall sensory quality of wine (Chidi, Bauer, and Rossouw, 2018). Consistent with the total acid and pH results above, the addition of UGJ also significantly increased the content of several organic acids in the low-acid wines. Fig. 1a showed that the addition of UGJ played a positive role in improving the total organic acid content of wine after the BW period. For example, in the AG-2 period, the total organic acid content in wine increased by 16.89 %-65.70 % compared with CK.

A total of 9 organic acids were detected in this study, as shown in Fig. 1b-d. Tartaric acid, malic acid and shikimic acid always existed and changed dynamically throughout the whole process of winemaking (Fig. 1b). Among them, the first two were the most important organic acids in wine, and the variation ranges in CK were 0.51–1.59 g/L and 0.66–1.70 g/L, respectively. The peak of shikimic acid occurred during the E-FM to DO period, with levels ranging from 0.81 to 1.45 g/L (Fig. 1). In general, with the progress of alcohol fermentation (PFM to E-FM), the contents of citric acid (Fig. 1d) and malic acid (Fig. 1b) decreased continuously, while those of succinic acid (Fig. 1c) increased continuously. This was mainly because succinic acid, citric acid and malic acid were intermediate products of the tricarboxylic acid cycle, which could be converted in turn during aerobic respiration. Succinic acid gradually decreased after the BW period and eventually could not be detected at AG-1, which might be due to succinic acid having the highest rate of esterification when compared with the other organic acids in wines (Lamikanra, 1997). As shown in Table S5, succinic acid was converted to diethyl succinate, ethyl isopentyl succinate, monoethyl succinate, etc., during aging. In addition, acetic acid (Fig. 1c) and fumaric acid were detected during PFM-BW and disappeared during AG-2. Oxalic acid was detected only after AG-1 (Fig. 1d).

As shown in Fig. 1b, the addition of UGJ significantly increased the content of tartaric acid in wine after the E-FM period (*p* < 0.05) and was positively correlated with the amount of UGJ in general. When wines were in the AG-2 period, the content of malic acid in the CK, UGJ4%, UGJ7% and UGJ8% groups decreased significantly, while the content of lactic acid increased significantly (*p* < 0.05). This might be due to the active physiological metabolism of malic acid, which was easily converted into lactic acid. At the same time, the decrease in free sulfur dioxide content during bottle storage led to the spontaneous initiation of malolactic-lactic fermentation during the wine’s aging period. However, the change trends were different in the different groups. The contents of malic acid and lactic acid in the other three groups were opposite to those in the above four groups during the AG-2 period. This might be due to the rich phenolic substances in UGJ, which had a complex effect on the lactic acid bacteria in wine, and the degree of influence depends on the type of bacterial strain and the type and concentration of phenolic substances (Devi and Anu-Appaiah, 2018).

In general, the addition of UGJ significantly increased the total amount of organic acids in wine, especially tartaric acid and malic acid, which increased by 13.89 %-59.03 % and 19.70 %-359.09 % in AG-2 compared with CK, respectively. What’s more, compared with CK, the addition of UGJ significantly reduced alcohol degree (Table S4). Therefore, UGJ has the potential to improve the deficiency of high sugar and low acidity in wines caused by global warming to some extent, thereby improving the balance and freshness of wine.

The addition of UGJ could also significantly reduce the volatile acidity (*p* < 0.05) (Table S4), excessive volatile acid has an obvious bitter taste and pungent vinegar taste, which are typical characteristics of wine corruption (Vilela-Moura et al., 2011). Thus, UGJ could effectively reduce the probability of wine spoilage, thereby reducing the use of SO_2_ during the winemaking process, consistent with previous studies (Fia, Bucalossi and Zanoni, 2021). In addition, when the addition of UGJ exceeded 2 %, the glycerinum content in wine was significantly reduced (*p* < 0.05). Therefore, maximizing the benefits of UGJ on the premise that the overall quality of wine was less affected and the amount of UGJ added was the key. For example, the UGJ2% and UGJ4% groups could not only significantly improve the low acidity and high alcohol content of wines, but also have little difference from CK in other physicochemical indexes.

### 3.2 Effects of UG on color and anthocyanins of low acid wine

#### 3.2.1 Colorimetric analysis

**Fig. 2:**
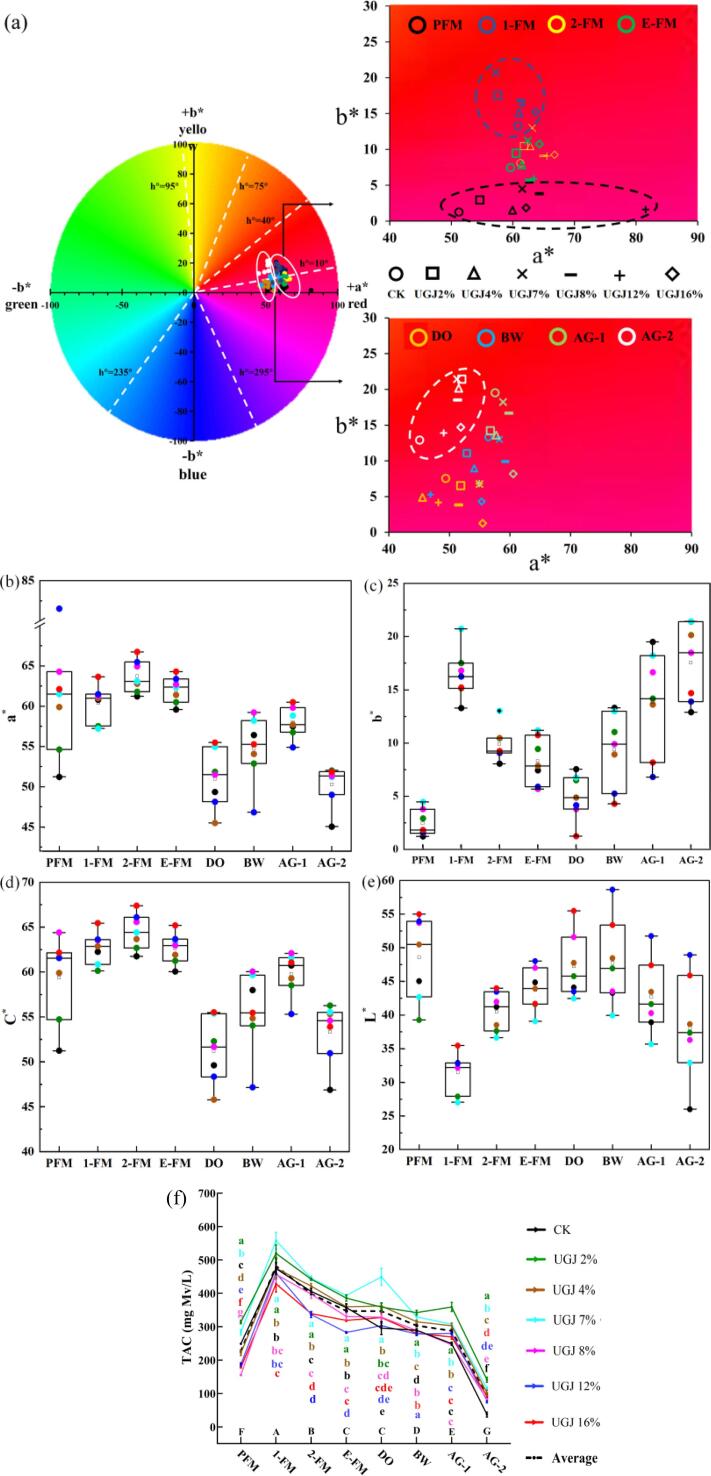
The colorimetric analysis of wines with different UGJ additions during the winemaking period (a) color distribution map; (b) a*; (c) b*; (d) C*; (e) L* and (f) total anthocyanin content (TAC).

The colorimetric analysis of wines during the winemaking period was presented in Fig. 2. As shown in Fig. 2a, the h° value of wines ranged from 0.80 to 23.95 throughout the winemaking period. With the extension of time, the wines gradually evolved from the purple zone to the red zone, and the specific color parameter trends were shown in Fig. 2b-e. The variation trends of the a* value and C* value was similar. During the DO period, the a* value and C* value dropped sharply, then rose and fell again with aging (Fig. 2a-b, d). The values of b* and L* fluctuated greatly during the winemaking period. The b* value was the lowest at PFM, increased sharply during the 1-FM period, decreased gradually, reached a valley value in the DO period and rose again (Fig. 2a, c), while the L* values showed the opposite trend (Fig. 2e). In general, it could be clearly seen from Fig. 2a that the red tone gradually weakened and the yellow tone gradually enhanced with the aging of wine, which was consistent with the results of previous studies (Wang, Liu, and Liu, 2018). Due to their unique 2-phenyl-benzylpyrilium structure, anthocyanins exhibit extremely high chemical activity and instability, resulting in complex changes in wine color during the aging process (Waterhouse and Zhu, 2020). For example, the pyranoid anthocyanins formed during the aging process contribute yellow or orange–red color to wine (Wang et al., 2018).

In addition, due to the addition of UGJ, the a*, b*, C* and L* values of wine in the AG-2 period were increased by 8.77 %-15.46 %, 7.78 %-66.00 %, 26.52 %-88.01 % and 8.69 %-20.02 %, respectively, compared to those of CK (Fig. 2). The results indicated that the addition of UGJ deepened the red and yellow tones of wine and enhanced the color saturation and brightness. The color variation of wines is jointly determined by free anthocyanins, copiganthocyanins and polymeric pigments (Heras-Roger, Alonso-Alonso, Gallo-Montesdeoca, Díaz-Romero, and Darias-Martín, 2016), and the factors responsible for this variation are very complex. For example, the abundant phenolic substances in UGJ (Table S3) affect the color characteristics of the wine through copigmentation. However, different copigments may produce different copigmentation; therefore, the wine showed different color characteristics due to different copigments (Teixeira et al., 2013). Moreover, the pH value could regulate the structural balance between different anthocyanins. When the pH value was low, the red and yellow color of wine deepened, and the brightness increased (Heras-Roger et al., 2016), which was consistent with the results obtained in this study. Therefore, it was hypothesized that the color change of wine caused by UGJ might be closely related to the decrease in pH value.

#### 3.2.2 Anthocyanins

The addition of UG significantly improved the problem of unstable color in low acid wines. The TAC in wines gradually increased before 1-FM, reached a peak of 428.43–558.13 mg/L (Fig. 2f) and then gradually decreased to 37.16–141.44 mg/L at AG-2. The significant reduction in anthocyanin content was mainly due to its poor stability. It was easily oxidized under the action of polyphenol oxidase and peroxidase or spontaneously degraded through deglycosylation by glycosidases (Lago-Vanzela et al., 2014). The addition of UGJ significantly increased the TAC content of low acid wine, it was observed from Fig. 2f that TAC in wine with different UGJ additions were significantly higher than those of CK (*p* < 0.05) at the AG-2 period. In particular, the TAC in the UGJ2% group was increased by 280.63 % compared with CK.

Five monomeric anthocyanins commonly found in wine include delphinidin-3-*O*-glucoside (Dp), cyanidin-3-*O*-glucoside (Cy), petunidin-3-*O*-glucoside (Pt), peonidin-3-*O*-glucoside (Pn) and malvidin-3-*O*-glucoside (Mv). They were very unstable during the aging process of wine and were prone to acetylation, coumarylation and caffeoylation along with aging (He et al., 2012). Table S6 suggested the changes in the nine monomeric anthocyanins during the winemaking period. Most of the monomeric anthocyanins decreased gradually after reaching the highest level due to maceration, which was consistent with the trend of TAC above (Fig. 2f). However, the contents of two coumarin acylated anthocyanins (peonidin-3-*O*-(*trans*-6-coumaryl)-glucoside (tPn-coum) and malvidin-3-*O*-(*trans*-6-coumaryl)-glucoside (tMv-coum)) were slightly different from those of other types, and the content of tMv-coum increased significantly in the AG-2 stage instead of decreasing continuously (*p* < 0.05), which proved that aging intensified the coumarylation of wine anthocyanins.

**Fig. 3:**
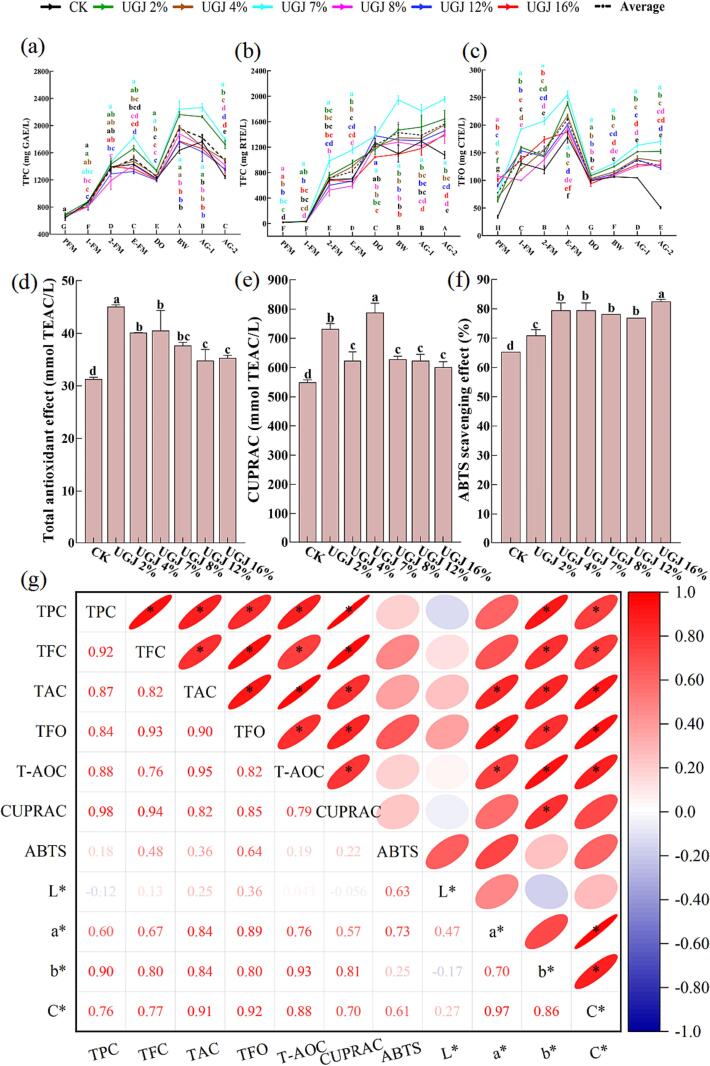
The analysis of phenolic substances in wines with different UGJ additions during the winemaking period and the antioxidant capacity in AG-2 period (a) total polyphenol content (TPC); (b) total flavonoids content (TFC) and (c) total flavan-3-ols content (TFO); (d) total antioxidant capacity (*T*-AOC); (e) copper ion reducing capacity (CUPRAC); (f) ABTS radical scavenging capacity (ABTS); (g) correlation analysis of phenolic substances, antioxidant capacity and color characteristics. Note: a. The different small letters indicate a significant difference among the different groups of wine (*p* < 0.05); b. The different capital letters indicate a significant difference among the different stages of wine (*p* < 0.05).

As shown in Table S6, Mv was the most abundant monomeric anthocyanin in wines, and its content ranged from 22.82 to 60.74 mg/L at AG-2, which was significantly the highest in UGJ 2 %. In general, the addition of UGJ significantly increased the content of noncoumarylated anthocyanins in wine, such as Dp and Cy, which might be because UGJ significantly increased the TPC, TFC and TFO in wine (Fig. 3). Phenolic substances such as flavonoids and phenolic acids could be used as copigments of anthocyanins, and their interaction could form a sandwich structure, which could play a copigmentation role in anthocyanins and improve the stability of monomeric anthocyanins (Gordillo et al., 2014).

### 3.3 Effects of UG on non-anthocyanin phenolic compounds and antioxidant activities of low acid wine

#### 3.3.1 Non-anthocyanin phenolic compounds

The addition of UG also significantly increased the phenolic substances of low-acid wine. Fig. 3 showed the dynamics of phenolic substances in wines with different UGJ additions during the winemaking period. From PFM to E-FM, both TPC (Fig. 3a) and TFO (Fig. 3c) indicated a fluctuating upward trend due to maceration. During the stabilization process from E-FM to DO, TPC and TFO form macromolecular complexes with proteins, polysaccharides, etc., which evolved until they became insoluble and precipitated from solution (Aleixandre-Tudo and du Toit, 2020). As a result, TPC and TFO in wine dropped significantly during the DO period. After DO, the TPC gradually increased and reached the highest value of 1704–2262.50 mg/L in the BW period and then gradually decreased. Since TPC was an overall evaluation of phenolic substances in wine, the late decline in TPC might be closely related to the decrease in total anthocyanins (Fig. 2f). Aside from CK, the TFO content in the other groups gradually increased after the DO period, which might be because UGJ contained a large amount of TFO, whose oligomers undergo complex polymerization-depolymerization under the mediation of acetaldehyde (Garcia-Falcon et al., 2007). As shown in Fig. 3b, the TFC of all groups suggested a fluctuating increase from the 1-FM to DO period. The TFC of the UGJ2%, UGJ4%, UGJ7% and UGJ16% groups increased slowly from the DO to AG-2 periods, while the other three groups were in dynamic equilibrium.

In addition, it was also observed from Fig. 3 that TPC, TFC and TFO in wine with different UGJ additions were significantly higher than those of CK (*p* < 0.05) at the AG-2 period. In particular, the TPC, TFC and TFO in the UGJ7% group were increased by 44.15 %, 81.60 % and 235.51 % compared with CK, respectively. This was mainly because UGJ contained rich phenolic substances, among which the TPC, TFC and TFO were 248.48 mg GAE/L, 40.78 mg RTE/L and 505.42 mg CTE/L, respectively (Table S3). However, wine is inherently a complex colloidal system, and the addition of different volumes of UGJ would also have different degrees of dilution effects on the must. Therefore, the content of phenolic substances in wine was not simply positively correlated with the amount of UGJ added.

#### 3.3.2 Antioxidant activities

Wine is an alcoholic beverage with notable health-related functions, mainly antioxidant, anti-aging, anti-diabetic, and anti-cardiovascular disease effects. The antioxidant capacity of wine at the AG-2 stage was shown in Fig. 3d-f 4a-c. Wine showed extremely strong antioxidant activity in three different antioxidant systems, and the *T*-AOC, CUPRAC and ABTS of CK were up to 31.29 mmol TEAC/L, 549.91 mmol TEAC/L and 65.39 %, respectively. Moreover, the addition of UGJ also increased the *T*-AOC, CUPRAC and ABTS by 11.18 %-44.00 %, 9.38 %-43.32 % and 8.50 %-26.14 %, respectively. The above results indicated that the addition of UGJ significantly enhanced the functional characteristics of low-acid wine, which was in line with the expectations of current consumers for functional alcoholic beverages.

To clarify the potential phytochemicals that contribute to the wine color and antioxidant capacity, the correlations between the antioxidant capacity, wine color and phenolic substances of the wine were analyzed, and the results were shown in Fig. 3g. As shown in Fig. 3g, the closer the absolute value of the correlation coefficient was to 1, the closer the shape of the graph was to an ellipse. The closer the correlation coefficient was to 1, the redder the color was. In contrast, the closer the correlation coefficient was to − 1, the bluer the color was.

Fig. 3g suggested that TPC (R_T-AOC_ = 0.88, R_CUPRAC_ = 0.98), TFC (R_T-AOC_ = 0.76, R_CUPRAC_ = 0.94), TAC (R_T-AOC_ = 0.95, R_CUPRAC_ = 0.82), and TFO (R_T-AOC_ = 0.82, R_CUPRAC_ = 0.85) were positively correlated with *T*-AOC and CUPRAC. This indicated that TPC, TFC, TAC and TFO all had prominent contributions to *T*-AOC and CUPRAC, and this result was consistent with previous studies (Lima, Ferreira, de Souza, Pereira, and Fedrigo, 2021). Furthermore, no significant correlation was observed between ABTS and the phenolic substances in this study, which might be because the ABTS was associated with multiple indicators, none of which was a major contributor. For the color parameters of wine, both the b* value and C* value was significantly positively correlated with TPC (R_b*_=0.90; R_C*_=0.76), TFC (R_b*_=0.80; R_C*_=0.77), TAC (R_b*_=0.84; R_C*_=0.91) and TFO (R_b*_=0.80; R_C*_=0.92), and the a* value was significantly positively correlated with TAC (R_a*_=0.84) and TFO (R_a*_=0.89). These results indicated that phenolic substances increased the red tone and color saturation of the wine on the one hand and led to the deepening of the yellow tone on the other hand, which further verified the conclusions drawn in Section 3.4 (Fig. 2).

### 3.4 Effects of UG on of sensory quality low acid wine

#### 3.4.1 Sensory evaluation

**Fig. 4:**
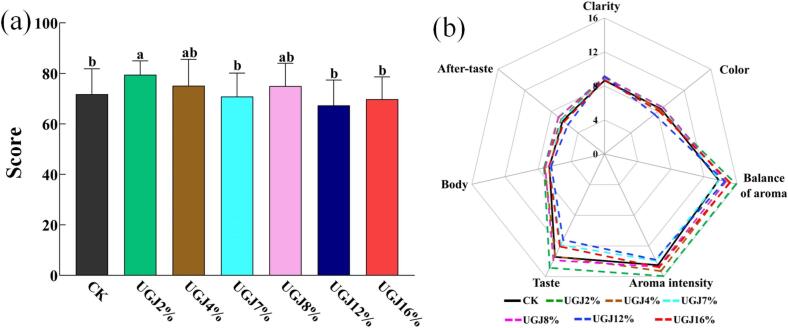
Sensory evaluation of wines with different UGJ additions in AG-2 period (a) total sensory ratings; (b) scores for different sensory attributes. Note: a. The different small letters indicate a significant difference among different groups (*p* < 0.05).

Fig. 4 showed the results of sensory evaluation in wines with different UGJ additions in the AG-2 period. As shown in Fig. 4a, the total score of sensory evaluation in the UGJ2% group was 78.29, which was significantly higher than that of CK (*p* < 0.05), while there was no significant difference between the other groups and CK (*p* > 0.05). This suggested that the addition of UGJ did not change the sensory quality of wine significantly, and the moderate addition had an improvement effect on the low-acid wines. As shown in Fig. 4b, the color score of UGJ7% was the highest. In terms of ‘balance of aroma’ and ‘aroma intensity’, UGJ2% scored 15.86 and 15.93, respectively. Moreover, the UGJ2% group also performed best in ‘body’ and ‘taste’, which might be because tartaric acid and malic acid increased by 13.89 % and 269.7 %, respectively, compared to those of CK (Fig. 1). Phenolic substances such as TPC, TFC, TFO were also significantly increased (Fig. 3). In this study, 2 % might be the most appropriate additive amount to improve the raw material for winemaking with high sugar and low acid, which could exactly supplement the freshness and complexity that the wine lacks and play a positive role in the sensory characteristics of wines with high sugar and low acidity. For ‘after-taste’, the scores of UGJ8% and UGJ2% were both 6.93, which was higher than the others, indicating that these wines had a longer aftertaste.

#### 3.4.2 VOCs

**Fig. 5:**
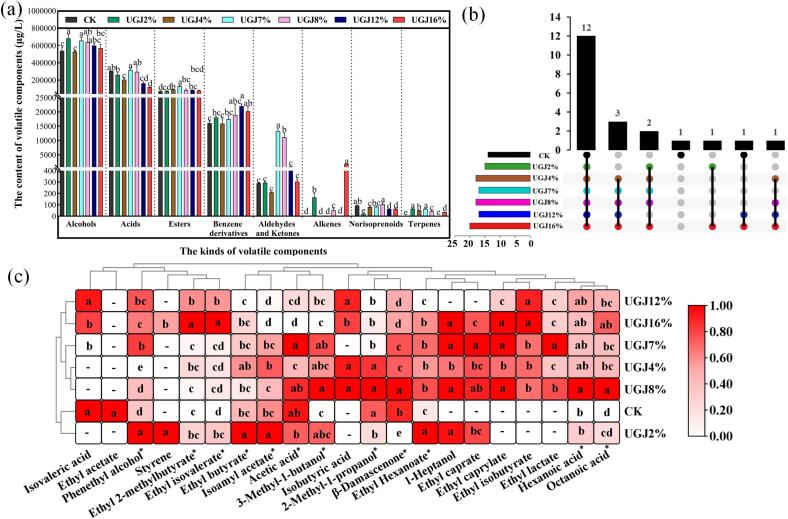
Volatile compounds analysis of wines with different UGJ additions in AG-2 period (a) total content of different volatile compounds; (b) venn diagram of key aroma compounds; (c) cluster heat map analysis of key aroma compounds. Note: a. The different small letters indicate a significant difference among different groups (*p* < 0.05); b. “—” indicates that OAV < 1, this volatile organic compound is not a key aroma compound; c. “*” indicates that this volatile organic compound is the key aroma compound in all groups of wines.

A wine’s aroma is created by a group of very complex volatile substances, and it is an important factor in wine style; the main aromas are varietal aroma, fermentation aroma produced in the process of alcohol fermentation and aging aroma evolved in the process of aging (Petronilho, Lopez, Ferreira, Coimbra, and Rocha, 2020). In this experiment, GC–MS was used to determine the VOCs of wines in AG-2, and 101 kinds of VOCs were detected. According to their functional groups, they were classified into 21 alcohols, 12 acids, 46 esters, 5 benzene derivatives, 11 aldehydes and ketones, 3 alkenes, 1 norisoprenoid and 2 terpenes, with total contents of 526897.07–681288.71 μg/L, 118712.43–316433.19 μg/L, 70771.24–129282.30 μg/L, 15921.98–22060.23 μg/L, 208.43–13255.73 μg/L, 0–1793.99 μg/L, 20.03–103.01 μg/L and 0–64.61 μg/L, respectively (Table S5). The variation coefficients of the various types of VOCs were calculated to understand the degree of dispersion among the groups. The variation coefficients of the above eight types of VOCs were 9.89 %, 32.38 %, 22.45 %, 12.08 %, 153.83 %, 232.14 %, 37.97 % and 73.91 %, respectively. The addition of UGJ had significant effects on different types of VOCs in wine, among which alkenes, aldehydes, ketones and terpenes were the most influential. Alkenes and terpenes mostly have pleasant smells, such as floral and fruity smells. Aldehydes and ketones were closely related to the ripeness of fruit and might give a green odor to the wine at higher concentrations (Lan et al., 2022). As shown in Fig. 5a, for aldehydes and ketones, the UGJ7% and UGJ8% groups were significantly higher than CK (*p* < 0.05), and there was no significant difference between the other groups and CK (*p* > 0.05). For alkenes, the UGJ16% and UGJ2% groups were significantly higher than the other groups (*p* < 0.05). For terpenes, the UGJ2% and UGJ7% groups were significantly higher than the other groups (*p* < 0.05). It was inferred that the UGJ2% and UGJ16% groups might have had more prominent flower and fruit aromas, the UGJ8% group might have had a more prominent green odor, and the UGJ7% group might have had more prominent flower and fruit aromas and a green odor.

However, the content of VOCs alone could not represent the contribution of odor, so the characteristic aromas of wine needed to be further investigated by key aroma compound analysis. A total of 21 key aroma compounds were detected in this study (Fig. 5), among which the CK, UGJ2%, UGJ4%, UGJ7%, UGJ8%, UGJ12% and UGJ16% groups contained 14, 15, 18, 17, 18, 17 and 20 species, respectively. Apparently, different UGJ additions increased the number of key aroma compounds in the wines to different degrees, which might have increased the complexity of the wine aromas. To further analyze the effects of different UGJ additions on wine aromas, a venn diagram (Fig. 5b) and cluster heatmap analysis (Fig. 5c) were performed on the key aroma compounds. According to Fig. 5b-c, 12 key aroma compounds were common to the seven groups of wines, including 3 alcohols, 3 acids, 5 esters and 1 norisoprenoid. There were 3 key aroma compounds in the UGJ4%, UGJ7%, UGJ8%, UGJ12% and UGJ16% groups, namely, ethyl caprylate, ethyl lactate and ethyl isobutyrate, which impart aromas of pineapple, apple and cream to the wine (Ferreira et al., 2000). Two key odor-active compounds, 1-heptanol and ethyl caprate, coexist in the UGJ2%, UGJ4%, UGJ7%, UGJ8% and UGJ16% groups, which contributed sweet pineapple and orange oil flavor to wine (Ferreira, López, and Cacho, 2000). Styrene was found only in the UGJ2% and UGJ16% groups and contributed floral aromas to the wine. Isovaleric acid existed in the CK, UGJ12% and UGJ16% groups, bringing a pungent sour odor to the wine (Lan et al., 2019). Isobutyric acid was found in the UGJ4%, UGJ8%, UGJ12% and UGJ16% groups, making wine fatty and rancid (Lan et al., 2019). In addition, ethyl acetate was a unique key aroma compound in CK, with a concentration of 13209.49 μg/L (Table S5). Studies have indicated that ethyl acetate might produce a varnish or nail polish odor when its concentration exceeds 12,000 μg/L and has an inhibitory effect on other VOCs (Ruiz et al., 2019), thus negatively affecting the sensory quality of CK.

As shown in Fig. 5c, the 7 groups of wines were divided into 2 categories, among which the first category was CK and UGJ2% (W1). The second category was composed of the other remaining groups (W2) and was further divided into 2 subgroups. The first subgroup was UGJ4%, UGJ7% and UGJ8% (W2-1). The second subgroup was UGJ12% and UGJ16% (W2-2), which roughly corresponded to the addition gradient of UGJ. For the key aroma compound common to all groups, the OAV of phenethyl alcohol, ethyl isovalerate, ethyl butyrate, isoamyl acetate, ethyl hexanoate was significantly increased in the UGJ2% group compared to CK (*p* < 0.05), which added pleasant floral and fruity aromas to wines such as apple, banana and rose (Ferreira et al., 2000). Meanwhile, the OAV of *β*-damascenone and 2-methyl-1-propanol decreased significantly in the UGJ2% group, *β*-damascenone had a strong rose aroma (Lan et al., 2022), and 2-methyl-1-propanol had a stimulating heteroalcohol odor (Ferreira, López, and Cacho, 2000). Thus, although the UGJ2% group had a weakened rose aroma, it also reduced the unpleasant odor brought by 2-methyl-1-propanol. In the W2-1 subgroup, 3-methyl-1-butanol, ethyl hexanoate and octanoic acid were increased to different degrees compared with CK (Fig. 5c) and were significantly the highest in the UGJ8% group. Ethyl hexanoate contributed to the aroma of fruits such as green apple and strawberry, but 3-methyl 1-butanol and octanoic acid produced heteroalcohol and unpleasant sweaty odors (Ferreira et al., 2000, Lilly et al., 2000), which negatively affected the wine senses. In addition, *β*-damascenone and hexanoic acid were also significantly highest in the UGJ8% group (*p* < 0.05), suggesting that although the fresh rose scent was significantly enhanced in the UGJ8% group, the unpleasant rancid was also significantly enhanced (Lan et al., 2019). In the W2-2 subgroup, the OAV of pleasant VOCs such as phenethyl alcohol and ethyl isovalerate were significantly increased, contributing more intense floral and fruity aromas to the wines. However, the OAV of ethyl 2-methylbutyrate and octanoic acid were also increased significantly compared to CK (*p* < 0.05), giving the UGJ12% and UGJ16% groups a more severe sweaty odor. Meanwhile, the OAV of isoamyl acetate, acetic acid and *β*-damascenone, which have pleasant odors, were also significantly decreased (*p* < 0.05).

Overall, the different UGJ additions increased the number of key aroma compounds in wine to varying degrees. In addition, pleasant VOCs such as phenethyl alcohol, ethyl hexanoate, styrene, and isoamyl acetate were significantly higher in the UGJ2% group than in the other groups, and unpleasant VOCs such as isovaleric acid and isobutyric acid were significantly lower. Therefore, the UGJ2% group not only retained the characteristic aroma of the original wine to the greatest extent but also significantly increased the strong floral and fruity aromas and avoided the rancidity of a large amount of vinegar, alcohol, and sweat odor caused by excessive amounts of UGJ. The UGJ4%, UGJ7%, UGJ8%, UGJ12% and UGJ16% groups, in addition to enhancing the rich aroma of apple, strawberry and rose, were also accompanied by strong ethanol, sweat odor and other stimulating and unpleasant tastes, especially the UGJ8% group, for which these tastes and smells were the most obvious. Based on the above findings, it could be concluded that adding 2 % UGJ could additionally contribute aroma complexity and intense floral and fruity aromas to the wine but also have on the premise of having less influence on the overall quality of the wine. This would further explain the reason why the UGJ2% group had the highest scores of ‘balances of aroma’ and ‘aroma intensity,’ as described in Section 3.6 “Sensory Evaluation” (Fig. 4b).

## 4 Conclusion

In conclusion, the addition of UGJ demonstrated potential to improve the deficiency of “high sugar and low acidity” of winemaking raw materials, thereby significantly improving the disadvantages of low acidity and high alcohol content of wine.; In especial the addition of 2 % UGJ also could significantly improve the sensory quality of the wine, contributing to the aromatic complexity and intense floral and fruity aromas of the wine. Therefore, UGJ addition has the potential for in-depth exploration and popularization. Previous studies showed that about 14436.16 kt worldwide of unripe grape was abandoned in orchards every year. If they're fully applied to the fermentation with “high sugar and low acid” wine grapes, about 721,808 kt of low acid wine will be improved globally. Meanwhile, fermentation technology will also turn thinned unripe grapes into higher-value items, promoting the green and sustainable development of the grape and wine industry.

## CRediT authorship contribution statement

**Mengyuan Wei:** Methodology, Investigation, Data curation, Visualization, Writing – original draft. **Yue Tian:** Methodology, Investigation, Data curation, Visualization, Writing – original draft. **Kekun Zhang:** Methodology, Investigation, Data curation. **Lei Wang:** Methodology, Investigation, Data curation. **Qian Ge:** Investigation, Data curation. **Tingting Ma:** Conceptualization, Writing – review & editing. **Yulin Fang:** Conceptualization, Methodology, Writing – review & editing. **Xiangyu Sun:** Conceptualization, Methodology, Writing – review & editing.

## Declaration of Competing Interest

The authors declare that they have no known competing financial interests or personal relationships that could have appeared to influence the work reported in this paper.

## Data Availability

Data will be made available on request.
