# Supplementary material for: Using abandoned unripe grape resources to solve the low-acid problem in the northwest wine region of China
Source: Food Chem X. 2023 Oct 31;20:100976. doi: 10.1016/j.fochx.2023.100976 (PMC10740056; doi:10.1016/j.fochx.2023.100976)
Supplement: Supplementary data 1 [file mmc1.docx]

Table S1. Sensory evaluation standards.

| Sensory attributes | | Standards | Score |
| --- | --- | --- | --- |
| Appearance | Clarity  (10) | Clear and transparent, no precipitation and suspended matter | 8-10 |
|  |  | A slight deposit or suspension | 5-7 |
|  |  | Obvious precipitates or suspended solids | 1-4 |
|  | Color  (10) | Purplish red, dark in color and glossy | 8-10 |
|  |  | Ruby red color, medium color, dark luster | 5-7 |
|  |  | Reddish brown, light color, loss of light | 1-4 |
| Aroma | Balance (20) | Fruit, wine balance, aroma is obvious, pure coordination | 16-20 |
|  |  | The aroma is more harmonious, fruit or wine aroma is weak, no difference | 9-15 |
|  |  | Fruity wine flavor is insufficient, fragrance is stronger, aroma is not correct | 1-8 |
|  | Intensity  (20) | Fruity bouquet, rich and elegant, soft and full | 16-20 |
|  |  | Average intensity, not lasting aroma | 9-15 |
|  |  | Heavy odor, basically no wine | 1-8 |
| Taste | Taste  (20) | Full - bodied, soft and refreshing, medium sweet and sour | 16-20 |
|  |  | Light bodied with good coordination and acceptable acidit | 9-15 |
|  |  | Less coordinated, low acid or sharp | 1-8 |
|  | Body  (10) | Complex, unique flavor, with typical, rich | 8-10 |
|  |  | Dark, typical and medium bodied | 5-7 |
|  |  | Monotonous, flat and unpleasant | 1-4 |
|  | After-taste  (10) | Long aftertaste | 8-10 |
|  |  | Medium and long | 5-7 |
|  |  | Shorter finish | 1-4 |

Table S2. Basic information on volatile compounds.

|  | Aroma component | CAS | Odor threshold* (μg/L) | Aroma description |
| --- | --- | --- | --- | --- |
|  |  |  |  |  |
| ***Alcohols*** | |  |  |  |
| 1 | 2-Methyl-1-propanol | 78-83-1 | 40000.00 | A choking, cough - provoking, nauseous smell, diluted with a mild sweet aroma |
| 2 | 4-Methyl-1-pentanol | 626-89-1 | — | — |
| 3 | 1-Hexanol | 111-27-3 | 8000 | A light green foliage, with hints of wine, fruit and fat |
| 4 | 3-Methyl-1-pentanol | 589-35-5 | 500 | Sharp fusel oil, wine and cocoa aromas with green fruit notes |
| 5 | Phenethyl alcohol | 22258.00 | 14000 | Sweet rose scent |
| 6 | 3-Methyl-1-butanol | 123-51-3 | 30000 | A heteroalcohol flavor and spicy taste, with mellow, ether, banana aroma |
| 7 | 3-Methylthiopropanol | 505-10-2 | — | Smell of fat/cabbage, garlic |
| 8 | 5-Nonanol | 623-93-8 | — | — |
| 9 | 4- Methyl -1-hexanol | 818-49-5 | — | — |
| 10 | 1-Nonanol | 143-08-8 | 600 | Strong sweet green rose wax and fruity fat wax aromas. |
| 11 | (S,S)-2,3-Butanediol | 19132-06-0 | 120000 | Creamy and fruity |
| 12 | 1-Butanol | 71-36-3 | 150000 | Alcohol, drugs, chemicals |
| 13 | 1-Heptanol | 111-70-6 | 1000 | Oily and spicy aromas, close to citrus |
| 14 | (S)-(+)-1,2-Propanediol | 4254-15-3 | — | — |
| 15 | Leaf alcohol | 928-96-1 | 400 | Fragrance of green, herb and green leaf |
| 16 | 3-Ethyl-2-pentanol | 597-49-9 | — | — |
| 17 | Cyclopentanol | 96-41-3 | — | — |
| 18 | 1-Undecanol | 112-42-5 | — | — |
| 19 | Trans-3-hexen-1-ol | 928-97-2 | 400 | — |
| 20 | Diisobutylcarbinol | 108-82-7 | — | — |
| 21 | Benzyl alcohol | 100-51-6 | 200000 | Sweet, floral, fruity aroma, fruity, cherry, fat flavor |
| ***Acids*** | |  |  |  |
| 22 | Acetic acid | 64-19-7 | 670 | — |
| 23 | Hexanoic acid | 142-62-1 | 420 | Rancid |
| 24 | Octanoic acid | 124-07-2 | 500 | Rancid |
| 25 | Decanoic acid | 334-48-5 | 1000 | Unpleasant fatty taste |
| 26 | Valeric acid | 109-52-4 | 3000 | — |
| 27 | DL-Phenylsuccinic acid | 635-51-8 | — | — |
| 28 | Isovaleric acid | 503-74-2 | 33 | Pungent rancidity, diluted with cheese, dairy, fruit aroma |
| 29 | 2-methylhexanoic acid | 4536-23-6 | — | — |
| 30 | 2-Oxobutyric acid | 600-18-0 | — | — |
| 31 | 2-Ethylheptanoic acid | 3274-29-1 | — | — |
| 32 | Isobutyric acid | 79-31-2 | 2300 | Lasting pungent smell, diluted with milk, sweet, cheese, fruit aroma |
| 33 | Butyric acid | 107-92-6 | 2500 | Rancid and cheesy |
| ***Esters*** | |  |  |  |
| ***Ethyl esters*** | |  |  |  |
| 34 | Palmitic acid ethyl ester | 628-97-7 | 1500 | Waxy, fruit, milk, cream, balm aroma, waxy, fruit, cream flavor |
| 35 | Ethyl Hexanoate | 123-66-0 | 14 | Green apple, strawberry, anise |
| 36 | Phenethyl acetate | 103-45-7 | 250 | Fruity, floral, honey and tropical fruit aromas diluted to 50mg/kg with honey, floral, fruity, citrus and yeast notes, light and not long lasting |
| 37 | Diethyl succinate | 123-25-1 | 120000 | Green, sweet and fruity, with hints of apple, pear and banana peel |
| 38 | Ethyl 2-methylbutyrate | 7452-79-1 | 18 | Fruit, green, grape aromas and aromas |
| 39 | Ethyl isovalerate | 108-64-5 | 3 | — |
| 40 | Ethyl butyrate | 105-54-4 | 20 | — |
| 41 | Ethyl caprate | 110-38-3 | 200 | Strong pineapple base, pineapple, banana, apple and other fruits, with a light rose nose, similar sweet taste |
| 42 | Ethyl laurate | 106-33-2 | 1500 | Grape |
| 43 | Ethyl acetate | 141-78-6 | 7500 | Sweet, fruity, and creamy |
| 44 | Ethyl caprylate | 106-32-1 | 147 | Ether aroma, sweet as pineapple fruit, and grape, cherry aroma |
| 45 | Ethyl lactate | 97-64-3 | 14000 | Sweet red currant, pear - like fruit, and light apple, banana, hyacinth, rose - like bouquet |
| 46 | Ethyl isobutyrate | 97-62-1 | 15 | The fragrance of wax, mold, fruit, cream, milk and sweet wine |
| 47 | Ethyl phenylacetate | 101-97-3 | 650 | Strong fruity, sweet orange nose with hints of fermented wine, herb, oily with hints of orange |
| 48 | Ethyl benzoate | 93-89-0 | 500 | Sweet, sour fruit aroma, diluted with creamy and pineapple notes |
| 49 | Ethyl isopentyl succinate | 28024-16-0 | — | — |
| 50 | Ethyl propionate | 105-37-3 | 1800 | Fragrance of flowers, fruit, powder, wood, animal, cocoa |
| 51 | Ethyl myristate | 124-06-1 | 500 | Sweet, fruity, medicated aroma and taste |
| 52 | Ethyldl-2-hydroxycaproate | 6946-90-3 | — | — |
| 53 | Monoethyl succinate | 1070-34-4 | — | — |
| 54 | Triethyl orthoformate | 122-51-0 | — | — |
| 55 | Ethyl heptanoate | 106-30-9 | 220 | Sweet, waxy and creamy flavors |
| 56 | Ethyl hex-2-enoate | 1552-67-6 | — | milk and cream |
| 57 | Ethyl 3-furancarboxylate | 614-98-2 | — | — |
| 58 | Ethyl hydrogen glutarate | 1070-34-4 | — | — |
| 59 | Ethyl isocyanoacetate | 2999-46-4 | — | — |
| 60 | Diethyl glutarate | 818-38-2 | — | — |
| ***Other esters*** | |  |  |  |
| 61 | Hexyl acetate | 142-92-7 | 1500 | — |
| 62 | Isobutyl acetate | 110-19-0 | 1600 | Fruity, green, waxy, Conneg, jackfruit, banana, strawberry flavor |
| 63 | Butyl acetate | 123-86-4 | 1600 | — |
| 64 | Isoamyl acetate | 123-92-2 | 30 | Banana, pear, apple |
| 65 | Caprylic acid methyl ester | 111-11-5 | 100 | Jackfruit aromas, spicy, sour and cheese |
| 66 | N-pentyl propionate | 624-54-4 | — | — |
| 67 | Gamma Butyrolactone | 96-48-0 | 20000 | Sweet fruit fragrance, green fragrance, fat fragrance, and cream, cheese |
| 68 | cis-3-Hexenyl lactate | 61931-81-5 | — | Wax fragrance, banana fragrance, sweet belt mixed green fragrance, taste like cream, fruity fragrance, fat wax rhyme |
| 69 | 2,2’-oxybisethyl dipropionate | 6942-59-2 | — | Pineapple, fruit, rum, garlic, meat, caramel aroma, fruit, garlic, caramel, rum flavor |
| 70 | 4-Hydroxybutanoic acid methyl ester | 925-57-5 | — | — |
| 71 | Isoamyl hexanoate | 2198-61-0 | — | — |
| 72 | n-Caprylic acid isobutyl ester | 5461-06-3 | — | — |
| 73 | Isoamyl octanoate | 2035-99-6 | — | — |
| 74 | N-capric acid isoamyl ester | 2306-91-4 | — | — |
| 75 | Allyl phenylacetate | 1797-74-6 | — | — |
| 75 | Linalyl butyrate | 78-36-4 | — | — |
| 77 | cis-3-Hexenyl formate | 33467-73-1 | — | — |
| 78 | Isoamyl lactate | 19329-89-6 | — | — |
| 79 | Isoamyl butyrate | 106-27-4 | — | — |
| ***Benzene derivatives*** | |  |  |  |
| 80 | 2,4,6-Tri-tert-butylphenol | 732-26-3 | — | — |
| 81 | 2,4-Di-tert-butylphenol | 96-76-4 | — | — |
| 82 | m-Xylene | 108-38-3 | — | — |
| 83 | Ethylbenzene | 100-41-4 | — | — |
| 84 | Toluene | 108-88-3 | — | — |
| ***Adehydes and ketones*** | |  |  |  |
| 85 | 2,4-Bis[(trimethylsilyl)oxy]benzaldehyde | 33617-38-8 | — | — |
| 86 | Formaldehyde | 50-00-0 | — | — |
| 87 | Acetal | 105-57-7 | — | — |
| 88 | 2,2-Dimethylhexanal | 996-12-3 | — | — |
| 89 | Acrolein | 107-02-8 | — | — |
| 90 | 1-Nonanal | 124-19-6 | 15 | Wax fragrance, citrus fragrance, fat fragrance, floral |
| 91 | Furfural | 98-01-1 | 15000 | Sweet, woody, breading, caramel, and baked |
| 92 | 3-Furaldehyde | 498-60-2 | — | — |
| 93 | 4’-Butoxyacetophenone | 5736-89-0 | — | — |
| 94 | Cyclobutyl methyl ketone | 3019-25-8 | — | — |
| 95 | 2,3-Pentanedione | 600-14-6 | — | Cream, caramel, nuts |
| ***Alkenes*** | |  |  |  |
| 96 | Cis-2,2-Dimethyl-3-hexene | 690-92-6 | — | — |
| 97 | Spiro [2,4] hepta-4,6-diene | 765-46-8 | — | — |
| 98 | Styrene | 100-42-5 | 80 | Resin, floral |
| ***Norisoprenoids*** | |  |  |  |
| 99 | beta-Damascenone | 23726-93-4 | 0.05 | A strong rose-like aroma |
|  | ***Terpenes*** |  |  |  |
| 100 | Alpha-Terpinene | 99-86-5 | — | — |
| 101 | Nerolidol | 7212-44-4 | 700 | Green, floral, woody, citrus, fruit aromas, mild apple notes |

Note: “-” represent not found.s ;* The odour thresholds were reference to the report (Li et al., 2023; Van Heimert, 2015).

Table S3. Analysis of physicochemical indexes in raw material.

|  | TSS  (°Brix) | pH | Reducing Sugars  (g/L) | TA  (g/L tartaric acid) | TPC | TTC | TAC | TFC | TFO |
| --- | --- | --- | --- | --- | --- | --- | --- | --- | --- |
| Cabernet Sauvignon | 27.37±0.12 | 3.30±0.1 | 255.50±4.95 | 5.10±0.09 | 48.26± 1.65  (mg GAE/g) | 28.22±1.23  (mg CTE/g) | 20.47±0.55  (mg Mv/g) | 13.09±0.34  (mg RTE/g) | 7.58±0.03  (mg CTE/g) |
| UGJ | 3.96±0 | 2.95±0 | 40.20±0.10 | 28.18±3.26 | 248.48±81.78  (mg GAE/L) | 162.94±7.61  (mg CTE/L) | — | 40.78±2.35  (mg RTE/L) | 505.42±10.62  (mg CTE/L) |

a. “UGJ” represents “unripe grape juice”; “TSS” represents “total soluble solids”; “TA” represents “titratable acid”; “TPC” represents “total polyphenol content”; “TTC” represents “total tannins content”; “TAC” represents “total anthocyanin content”; “TFC” represents “total flavonoid content”; “TFO” represents “total flavan-3-ols content”.

Table S4. Analysis of physicochemical indexes in wines with different UGJ additions in BW period.

| Analytical data | CK | UGJ2% | UGJ4% | UGJ7% | UGJ8% | UGJ12% | UGJ16% |
| --- | --- | --- | --- | --- | --- | --- | --- |
| pH | 3.66±0a | 3.64±0b | 3.57±0c | 3.54±0d | 3.44±0e | 3.34±0f | 3.25±0g |
| TA (g/L) | 5.88±0e | 6.06±0.18e | 6.38±0.04d | 6.68±0.11c | 6.74±0.24c | 7.51±0.19b | 8.12±0.19a |
| Alcohol degree (%vol) | 14.80±0.05a | 14.50±0.24b | 14.36±0.18c | 13.81±0.21d | 10.03±0.52g | 12.94±0.48e | 12.66±0.11f |
| Volatile acidity (g/L) | 0.19±0.01a | 0.18±0b | 0.16±0c | 0.16±0.01c | 0.13±0e | 0.14±0.01d | 0.12±0f |
| Glucose (g/L) | 2.18±0.02c | 2.37±0.01bc | 2.28±0bc | 2.48±0b | 2.72±0.01a | 2.44±0.21b | 2.76±0.01a |
| Fructose (g/L) | 1.28±0.01e | 1.46±0.01de | 1.53±0.01d | 1.78±0.01c | 2.11±0.02b | 2.36±0.23a | 2.43±0a |
| Glycerinum (g/L) | 10.63±0.1a | 10.37±0.03abc | 10.15±0.01bc | 10.04±0bc | 10.09±0bc | 9.83±0.83c | 9.00±0d |

a. The different small letters indicate a significant difference (*p*<0.05) among different groups. b. “TA” represents “titratable acid”; “UGJ” represents “unripe grape juice”.

Table S5 The content of volatile compounds in wines with different UGJ additions in AG-2 period.

|  | Aroma component | Content/(μg/L) | | | | | | |
| --- | --- | --- | --- | --- | --- | --- | --- | --- |
|  |  | CK | UGJ2% | UGJ4% | UGJ7% | UGJ8% | UGJ12% | UGJ16% |
| ***Alcohols*** | |  |  |  |  |  |  |  |
| 1 | 2-Methyl-1-propanol | 53153.06±5196.31a | 42136.09±2809.02b | 55580.83±1992.3a | 38376.2±2996.71b | 58810.47±6273.54a | 37512.68±1380.45b | 40197.27±8524.75b |
| 2 | 4-Methyl-1-pentanol | 109.48±15.13b | 130.95±5.2ab | 123.09±3.73ab | 124.36±13.23ab | 139.25±28.53a | 115.86±4.09ab | 105.53±1.35b |
| 3 | 1-Hexanol | 2274.71±144.38ab | 2361.36±167.54a | 1997.96±10.56bc | 2263.96±172.28ab | 1902.69±342.45cd | 1636.11±57.05d | 1301.07±31.96e |
| 4 | 3-Methyl-1-pentanol | 92.11±13.43bc | 151.91±11.75a | 133.5±6.09ab | 137.01±18.84ab | 154.31±56.63a | 102.13±11.04bc | 71.44±3.63c |
| 5 | Phenethyl alcohol | 108761.08±9091.59d | 211511.12±15138.17a | 55496.91±368.7e | 184498.01±6607.9b | 120456.37±23657.37d | 173603.77±14222.97bc | 156420.9±6733.99c |
| 6 | 3-Methyl-1-butanol | 369668.74±26537.05c | 422678.21±28434.58abc | 411713.66±3945.81abc | 426971.33±29039.76ab | 443741.25±50896.13a | 384066.33±8143.21bc | 368303.67±29324.54c |
| 7 | 3-Methylthiopropanol | 143.89±18.96bcd | 170.17±9.68ab | 153.11±11.79bc | 192.04±3.92a | 166.02±31.6ab | 129.65±14.91cd | 115.95±2.45d |
| 8 | 5-Nonanol | 41.34±0.66b | 50.47±0.67a | 40.05±2.42b | 41.95±4.21b | 38.22±2.49b | ND | ND |
| 9 | 4- Methyl -1-hexanol | 3064.3±25.52a | ND | ND | ND | ND | ND | ND |
| 10 | 1-Nonanol | 247.09±39.87a | ND | ND | ND | ND | 166.48±0.03b | ND |
| 11 | (S,S)-2,3-Butanediol | 91.94±25.63a | ND | ND | ND | 101.12±45.21a | 98.01±28.74a | ND |
| 12 | 1-Butanol | 212.54±9.01a | ND | 161.36±8.63b | 151.18±5.43bc | 143.2±17.19c | ND | ND |
| 13 | 1-Heptanol | ND | 2028.87±25.22a | 1408.56±190.03b | 2111.76±122.72a | 2101.73±262.05a | 650.29±7.94c | 2127.99±5.38a |
| 14 | (S)-(+)-1,2-Propanediol | ND | ND | 81.56±17.62a | ND | ND | ND | ND |
| 15 | Leaf alcohol | ND | ND | ND | ND | 68.2±12.07a | ND | ND |
| 16 | 3-Ethyl-2-pentanol | ND | ND | ND | ND | 164.95±46.85a | ND | 36.47±1.02b |
| 17 | Cyclopentanol | ND | ND | ND | ND | 8109.38±877.82a | ND | ND |
| 18 | 1-Undecanol | ND | ND | ND | ND | 142.7±41.08a | 85.57±0.09b | 147.43±1.73a |
| 19 | Trans-3-hexen-1-ol | ND | ND | ND | ND | ND | 56.14±2.85a | ND |
| 20 | Diisobutylcarbinol | ND | ND | ND | ND | ND | 60.08±4.38a | ND |
| 21 | Benzyl alcohol | ND | ND | ND | 519.19±18.39a | 381.45±69.43b | 247.34±8.82c | 195.76±10.03d |
| **Number** | | 12 | 9 | 11 | 11 | 16 | 14 | 11 |
| **Subtotal** | | 537863.16±40985.36c | 681288.71±46596.68a | 526897.07±6536.97c | 655396.64±39004.47a | 636632.2±80300.71ab | 598539.5±23886.36abc | 569027.55±44626.4bc |
| ***Acids*** | |  |  |  |  |  |  |  |
| 22 | Acetic acid | 300700.75±2175.04ab | 256657.75±23699.72b | 198472.84±12608.33c | 311827.12±13585.97a | 287338.63±67531.96ab | 154834.87±18008.05cd | 110419.59±1916.44d |
| 23 | Hexanoic acid | 1043.55±92.91b | 1145.96±88.29b | 1184.37±1.73ab | 1181.48±64.76ab | 1379.74±247.54a | 1223.86±76.73ab | 1181.53±57.05ab |
| 24 | Octanoic acid | 977.71±35.07d | 1128.36±12.9cd | 1267.27±49.53bc | 1247.5±47.5bc | 1650.46±293.15a | 1289.58±60.81bc | 1470.93±63.15ab |
| 25 | Decanoic acid | 135.59±47.2b | 146.24±24.09b | 140.35±14.68b | 146.65±2.25b | 254.15±18.35a | 110.07±0.29b | 147.67±2.21b |
| 26 | Valeric acid | 420.5±380.3c | 1419.67±9.2b | 83.03±40.04d | 1907.75±123.04a | ND | ND | ND |
| 27 | DL-Phenylsuccinic acid | 175.28±73.77a | ND | 102.12±0.12bc | 122.7±8.22b | ND | 66.61±3.34c | ND |
| 28 | Isovaleric acid | 1460.86±171.57a | ND | ND | ND | ND | 1364.2±82.03a | 1187.09±14.58b |
| 29 | 2-methylhexanoic acid | ND | 402.73±29.07a | 375.16±3.07b | ND | ND | ND | ND |
| 30 | 2-Oxobutyric acid | ND | 216.92±10.08a | ND | ND | ND | ND | ND |
| 31 | 2-Ethylheptanoic acid | ND | 44.3±12.8a | ND | ND | ND | ND | ND |
| 32 | Isobutyric acid | ND | 77.85±0.7c | 3423.15±1.37a | ND | 3672.94±485.46a | 3345.71±157.59a | 2987.81±23.49b |
| 33 | Butyric acid | ND | ND | ND | ND | 1685.7±358.55a | 38.64±2.11c | 1317.81±53.11b |
| **Number** | | 7 | 9 | 8 | 6 | 5 | 7 | 6 |
| **Subtotal** | | 304914.23±1903.17ab | 261239.78±23606.18b | 205048.29±12510.13c | 316433.19±13810.8a | 295981.62±68217.91ab | 162273.53±18384.26cd | 118712.43±1842.65d |
| ***Esters*** | |  |  |  |  |  |  |  |
| ***Ethyl esters*** | |  |  |  |  |  |  |  |
| 34 | Palmitic acid ethyl ester | 35.47±24.62b | 74.75±46.41ab | 77.26±30.67ab | 92.07±6.94a | 99.35±8.29a | 63.95±1.47ab | 43.71±6.05b |
| 35 | Ethyl Hexanoate | 157.16±5.96c | 274.59±16.94a | 215.11±21.82b | 232.27±19.45b | 242.88±23.26b | 137.87±5.09c | 218.38±17.22b |
| 36 | Phenethyl acetate | 93.51±2.32a | 98.91±7.37a | 89.19±1.66ab | 66.14±5.78c | 78.4±13.82b | 51.87±2.97d | 46.82±3.35d |
| 37 | Diethyl succinate | 2639.55±209.45c | 4731.79±404.47b | 4729.9±22.86b | 4299.52±297.47b | 5661.98±1077.49a | 6450.68±336.1a | 3863.25±151.5b |
| 38 | Ethyl 2-methylbutyrate | 28.77±2.07c | 33.08±5.16bc | 32.51±2.7bc | 29.96±1.93c | 29.54±3.05c | 38.23±0.02b | 44.66±5.94a |
| 39 | Ethyl isovalerate | 32.02±1.58d | 39.2±1.91bc | 35.35±2.86cd | 33.89±1.81cd | 34.91±3.69cd | 43.64±0.99b | 52.5±7.7a |
| 40 | Ethyl butyrate | 136.63±7.43bc | 162.6±15.27a | 142.94±11.55ab | 131.34±10.81bc | 122.57±4.06bc | 115.95±6.17c | 128.2±17.8bc |
| 41 | Ethyl caprate | 163.63±2.24d | 348.37±0.75bc | 334.41±57.68bc | 436.99±19.58a | 394.44±66.31ab | 175.54±3.63d | 328.63±9.7c |
| 42 | Ethyl laurate | 71.82±0.12e | 77.57±0.05ab | 75.93±1.29bc | 79.02±0.29a | 78.78±2.34a | 73.2±0.07de | 74.35±0.41cd |
| 43 | Ethyl acetate | 13209.49±1083.68a | 5983.45±622.6b | 2520.75±214.61d | 4748.08±202.6c | 4991.75±1055.22bc | 4516.78±12.37c | 3256.59±482.51d |
| 44 | Ethyl caprylate | 0.02±0d | 0.04±0d | 387.67±50.37b | 570.11±32.11a | 565.75±64.97a | 181.09±10.45c | 568.03±0.42a |
| 45 | Ethyl lactate | ND | ND | 29624.9±1072.71c | 81145.77±4736.81a | 62967.56±13062.61b | 23942.83±1699.6c | 24453.86±608.18c |
| 46 | Ethyl isobutyrate | ND | ND | 17.29±1.34b | 16.74±0.83b | 16.86±1.37b | 22.52±0.98a | 24.89±3.54a |
| 47 | Ethyl phenylacetate | 77.79±11.5c | 78.21±3.36c | 73.05±5.53c | 319.08±7.51b | 346.98±34.29a | 370.43±10.11a | 364.98±15.18a |
| 48 | Ethyl benzoate | 314.86±224.09a | 214.48±118.82ab | 132.9±62.99ab | 109.48±40.86b | 95.48±26.36b | 71.32±19.39b | 52.78±19.38b |
| 49 | Ethyl isopentyl succinate | 269.02±26.56d | 342.81±41.62cd | 360.59±8.35c | 394.95±34.7c | 493.6±102.38ab | 502.37±23.39a | 412.58±31.27bc |
| 50 | Ethyl propionate | 269.2±20.84a | 269.44±20.61a | 231.96±16.02abc | 243.53±17.66ab | 202.48±10.52c | 218.55±14.04bc | 224.42±36.01bc |
| 51 | Ethyl myristate | 87.7±17.27e | 324.23±2.18b | 201.93±2.75d | 414.82±28.15a | 254.01±55.97c | 173.67±27.61d | 124.54±4.86e |
| 52 | Ethyldl-2-hydroxycaproate | 116.48±5.39c | ND | 136.16±2.17b | ND | 159.2±26.39a | ND | 164.7±3.54a |
| 53 | Monoethyl succinate | 1085.05±392.21ab | 1373.73±48.06a | 908.26±148b | 1025.82±228.83ab | 1422.08±117.96a | 1155.17±243.8ab | 917.28±114.86b |
| 54 | Triethyl orthoformate | 1408.24±154.66a | ND | ND | ND | ND | ND | ND |
| 55 | Ethyl heptanoate | 76.3±0.17cd | 107.27±0.74a | 77.03±7.08 | 97.25±3.47b | 81.47±6.72c | 52.26±2.28e | 69.57±0.18d |
| 56 | Ethyl hex-2-enoate | ND | 93.62±2.26a | ND | 83.18±4.12b | ND | 53.11±0.07c | 53.14±0.25c |
| 57 | Ethyl 3-furancarboxylate | ND | ND | 28.92±0.05c | 29.17±2.69c | ND | 54.42±2.32b | 61.19±0.9a |
| 58 | Ethyl hydrogen glutarate | ND | ND | 10.22±0.2a | ND | ND | ND | ND |
| 59 | Ethyl isocyanoacetate | ND | ND | ND | ND | ND | 123.4±5.31a | ND |
| 60 | Diethyl glutarate | ND | ND | ND | ND | ND | ND | 16.93±1a |
| ***Other esters*** | |  |  |  |  |  |  |  |
| 61 | Hexyl acetate | 5.54±0.04c | 7.06±0.08a | 5.79±0.18b | 5.14±0.2d | 4.49±0.23e | 2.7±0.06g | 3.26±0.03f |
| 62 | Isobutyl acetate | 58.45±0.44b | 72.88±3.71a | 51.56±3.48c | 56.05±2.5bc | 45.74±4.3d | 44.43±1.47d | 39.19±3.45e |
| 63 | Butyl acetate | 42.93±0.39a | ND | ND | ND | ND | ND | ND |
| 64 | Isoamyl acetate | 6053.84±206.23bc | 7168.67±364.54a | 6427.66±443.1b | 5872.38±257.37bc | 5566.85±377.96c | 4512.76±78.12d | 4393.25±480.92d |
| 65 | Caprylic acid methyl ester | ND | ND | 1.19±0.07c | 1.56±0.05a | 1.38±0.1b | ND | 1.4±0.01b |
| 66 | N-pentyl propionate | 43947±3076.59b | 50171.82±3310.73a | 43491.39±426.25b | 27748.88±1949.84c | 7.24±1.83d | 40620.96±876.06b | ND |
| 67 | Gamma Butyrolactone | 95.23±9.06b | 114.8±3.21a | 85.44±5.68b | 93.78±2.55b | 86.82±13.56b | 59.54±6.98c | 48.44±0.28c |
| 68 | cis-3-Hexenyl lactate | 7.85±0.29b | ND | ND | 21.02±0.49a | ND | ND | ND |
| 69 | 2,2’-oxybisethyl dipropionate | 71.88±5.75a | 27.07±4.29c | ND | 28.04±4.52c | ND | 44.35±0.84b | 32.62±3.35c |
| 70 | 4-Hydroxybutanoic acid methyl ester | 215.82±41.14a | ND | 162.37±19.32b | 152.56±3.87b | 111.79±22.86c | 66.53±16.33d | ND |
| 71 | Isoamyl hexanoate | ND | 236.91±1.48a | 106.78±106.78b | ND | 260.06±50.95a | ND | ND |
| 72 | n-Caprylic acid isobutyl ester | ND | 22.84±0.26a | 14.01±2.53b | ND | 25.33±6.95a | ND | ND |
| 73 | Isoamyl octanoate | ND | 514.47±40.06a | 329.47±62.91b | 491.47±4.09a | 500.39±131.74a | 166.48±0.03c | 497.49±30.41a |
| 74 | N-capric acid isoamyl ester | ND | 145.15±2.19a | 96.61±20.1b | 140.86±11.69a | ND | ND | ND |
| 75 | Allyl phenylacetate | ND | ND | ND | 22.42±0.7a | ND | ND | ND |
| 75 | Linalyl butyrate | ND | ND | ND | 48.97±4.05a | ND | ND | ND |
| 77 | cis-3-Hexenyl formate | ND | ND | ND | ND | 29.52±4.49a | 22.93±1.13b | ND |
| 78 | Isoamyl lactate | ND | ND | ND | ND | 209.26±39.35a | ND | ND |
| 79 | Isoamyl butyrate | ND | ND | ND | ND | ND | ND | 38967.89±3068.63a |
| Number | | 29 | 29 | 34 | 35 | 34 | 33 | 33 |
| Subtotal | | 70771.24±5059.7d | 73109.8±4754.62cd | 91216.48±2244.63b | 129282.3±7848.68a | 85278.04±12648.98bc | 84232.06±3310.28bc | 79631.22±4761.74bcd |
| ***Volatile phenols*** | |  |  |  |  |  |  |  |
| 80 | 2,4,6-Tri-tert-butylphenol | 427.59±13.44ab | 484.36±9.41a | 291.46±71.52c | 315.22±22.01c | 182.1±44.13d | 413.27±8.35b | 282.21±14.2c |
| 81 | 2,4-Di-tert-butylphenol | 15373.91±742.24c | 17279.54±514.12bc | 15469.5±1801.83c | 16812.57±1213.28bc | 18721.07±3672.85abc | 21646.96±698.31a | 19774.49±1673.59ab |
| 82 | m-Xylene | 113.73±3.78b | 147.79±0.11a | 76.84±27.02c | 74.17±1.68c | ND | ND | ND |
| 83 | Ethylbenzene | 138.31±6.55a | ND | ND | ND | ND | ND | 80.82±2.92b |
| 84 | Toluene | ND | 157.58±42.77b | 84.18±24.81c | 224.42±13.56a | ND | ND | 50.38±6.2d |
| Number | | 4 | 4 | 4 | 4 | 2 | 2 | 4 |
| Subtotal | | 16053.54±739.13c | 18069.28±507.37bc | 15921.98±1678.48c | 17426.38±1250.54bc | 18903.17±3628.71abc | 22060.23±706.66a | 20187.89±1684.52ab |
| ***Adehydes and ketones*** | |  |  |  |  |  |  |  |
| 85 | 2,4-Bis[(trimethylsilyl)oxy]benzaldehyde | ND | ND | 70.27±1.17a | 66.35±6.57a | ND | ND | 47.36±1.7b |
| 86 | Formaldehyde | ND | ND | ND | 13026.64±874.32a | ND | ND | ND |
| 87 | Acetal | ND | ND | ND | ND | 2253.02±62.68a | ND | ND |
| 88 | 2,2-Dimethylhexanal | ND | ND | ND | ND | 8110.61±859.48a | ND | ND |
| 89 | Acrolein | ND | ND | ND | ND | 726.95±635.66a | ND | ND |
| 90 | 1-Nonanal | 2.47±0.21a | ND | ND | ND | ND | 2.88±0.99a | ND |
| 91 | Furfural | 286.83±2.28b | 294.99±6.45a | ND | ND | 63.34±0.85d | 204.58±7.62c | ND |
| 92 | 3-Furaldehyde | ND | ND | 138.16±5.22a | 105.88±5.82b | ND | ND | 110.99±3.64b |
| 93 | 4’-Butoxyacetophenone | ND | ND | ND | 56.86±3.35a | ND | 23.48±2.49b | ND |
| 94 | Cyclobutyl methyl ketone | ND | ND | ND | ND | ND | 649.42±9.92a | ND |
| 95 | 2,3-Pentanedione | ND | ND | ND | ND | ND | ND | 145.7±15.97a |
| Number | | 2 | 1 | 2 | 4 | 4 | 4 | 3 |
| Subtotal | | 289.3±2.07c | 294.99±6.45c | 208.43±4.05c | 13255.73±890.06a | 11153.92±1431.61b | 880.37±21.02c | 304.05±21.31c |
| ***Alkenes*** | |  |  |  |  |  |  |  |
| 96 | Cis-2,2-Dimethyl-3-hexene | ND | ND | ND | ND | ND | ND | 1705.01±3.25a |
| 97 | Spiro[2,4]hepta-4,6-diene | ND | ND | ND | ND | ND | ND | ND |
| 98 | Styrene | 0c | 165.32±38.75a | ND | ND | ND | ND | 88.98±1.68b |
| Number | | 0 | 1 | 0 | 0 | 0 | 0 | 2 |
| Subtotal | | ND | 165.32±38.75b | ND | ND | 52.35±14.28c | ND | 1793.99±1.57a |
| ***Norisoprenoids*** | |  |  |  |  |  |  |  |
| 99 | beta-Damascenone | 93.99±5.81ab | 20.03±1.25e | 80.37±1.91c | 83.51±6.18bc | 103.01±13.56a | 61.92±1.62d | 63.39±3.22d |
| Number | | 1 | 1 | 1 | 1 | 1 | 1 | 1 |
| Subtotal | | 93.99±5.81ab | 20.03±1.25e | 80.37±1.91c | 83.51±6.18bc | 103.01±13.56a | 61.92±1.62d | 63.39±3.22d |
| ***Terpenes*** | |  |  |  |  |  |  |  |
| 100 | Alpha-Terpinene | ND | 64.61±4.1a | 50.56±2.22b | 55.08±3.63b | 42.75±4.1c | ND | 36.09±0.94d |
| 101 | Nerolidol | ND | ND | ND | 9.2±0.48a | ND | ND | ND |
| Number | | 0 | 1 | 1 | 2 | 1 | 0 | 1 |
| Subtotal | | ND | 64.61±4.1a | 50.56±2.22b | 64.27±4.11a | 42.75±4.1c | ND | 36.09±0.94d |
| Total Number | | 57 | 56 | 60 | 64 | 66 | 63 | 63 |
| Total | | 929985.46±4721.99bc | 1034187.91±2822.44ab | 839372.62±1961.39cd | 1131877.76±6281.73a | 1048104.31±16336.99ab | 868047.61±4631.2cd | 789720.53±4588.09d |

a. The different small letters indicate a significant difference (*p*<0.05) among different groups. b. “ND” represents “Not Detected”; “UGJ” represents “unripe grape juice”.

Table S6. The content of anthocyanin in wines with different UGJ additions during the winemaking period.

| Treatments | | Dp (mg/L) | Cy (mg/L) | Pt (mg/L) | Pn (mg/L) | Mv (mg/L) | Pn-acet (mg/L) | Mv-acet (mg/L) | tPn-coum (mg/L) | tMv-coum (mg/L) |
| --- | --- | --- | --- | --- | --- | --- | --- | --- | --- | --- |
| PFM | CK | 29.61±0.55cC | 9.85±0.19dA | 35.89±0.05cE | 46.23±0.57bA | 421.36±5.88cF | 8.25±0.13cC | 122.82±0.51dE | 1.74±0.07dD | 19.18±1.29cF |
|  | UGJ 2% | 41.98±1.21bC | 13.09±0.29bA | 42.82±1.24bF | 57.36±1.39aA | 464.73±10.66aF | 9.58±0.23bD | 142.58±2.12bE | 2.85±0.93bD | 28.04±4.5bF |
|  | UGJ 4% | 42.16±1.59bB | 10.76±0.43cA | 35.01±2.38cF | 37.55±2.12cA | 307.39±17.35dF | 9.42±0.79bD | 131.68±9.8cE | 2.51±0.9bcD | 22.22±5cG |
|  | UGJ 7% | 63.56±0.87aB | 14.62±0.22aA | 51.20±0.77aE | 56.06±0.86aA | 438.67±5.47bF | 11.99±0.29aF | 172.75±3.2aF | 4.28±0.46aC | 36.96±3.34aE |
|  | UGJ 8% | 41.61±1.06bC | 9.62±0.19dA | 31.26±0.85dF | 31.26±0.83dC | 244.96±5.54fF | 8.67±0.31cD | 114.38±3.29eF | 2.20±0.2bcdF | 19.40±1.72cF |
|  | UGJ 12% | 41.45±1.33bC | 9.57±0.4dA | 31.76±0.97dF | 32.10±0.9dB | 260.83±5.95eF | 8.55±0.45cE | 116.20±4.75eF | 2.01±0.29cdE | 18.46±2.79cF |
|  | UGJ 16% | 42.36±0.44bC | 9.84±0.08dA | 32.20±0.46dE | 31.69±0.44dB | 252.19±3.14efF | 8.74±0.23cC | 115.72±2.36eE | 1.90±0.23cdE | 20.25±1.69cF |
| 1-FM | CK | 75.89±1.48eA | 3.41±0.04fD | 82.22±1.22cA | 30.67±0.29gB | 726.36±7.18bA | 22.71±0.31dA | 290.05±3.04dA | 8.78±0.19dA | 83.44±2.11dA |
|  | UGJ 2% | 82.49±0.86cA | 4.11±0.04dC | 85.40±0.69bA | 36.02±0.1eB | 757.29±4.27aB | 24.58±0.18bA | 311.62±2.04bA | 10.39±0.18bA | 97.31±1.7bA |
|  | UGJ 4% | 78.09±1.14dA | 4.04±0.03eC | 77.84±0.86eA | 35.00±0.34fB | 674.78±5.45cA | 22.96±0.38dA | 289.28±3.09dA | 9.46±0.32cA | 88.13±2.87cA |
|  | UGJ 7% | 90.45±0.61aA | 5.22±0.06aC | 87.40±0.42aA | 44.82±0.27aB | 761.40±3.41aB | 26.38±0.39aA | 333.45±2.29aA | 11.04±0.15aA | 104.22±1.82aA |
|  | UGJ 8% | 86.35±1.89bA | 4.95±0.07bB | 80.25±1.39dA | 40.13±0.43bA | 656.23±7.81dA | 24.01±0.04cA | 297.08±3.32cA | 10.54±0.3bA | 95.76±2.67bA |
|  | UGJ 12% | 76.81±0.21deA | 4.52±0.03cB | 72.86±0.66fA | 37.19±0.2cA | 616.27±4.66eA | 22.20±0.06eA | 277.56±2.29eA | 9.43±0.23cA | 85.35±2.3dA |
|  | UGJ 16% | 75.75±0.76eA | 0.71±0gG | 69.06±0.52gA | 36.51±0.29dA | 563.41±3.39fA | 21.26±0.23fA | 261.42±2.02fA | 9.26±0.19cA | 84.64±1.79dA |
| 2-FM | CK | 34.97±1.01eB | 4.31±0bC | 58.42±0.88eB | 24.08±0.36fC | 695.64±3.97cB | 16.94±0.09eB | 248.65±2.25dB | 4.01±0.14dB | 51.33±1.78eB |
|  | UGJ 2% | 46.93±1.07cB | 3.91±3.06bC | 71.09±1.08bB | 29.82±0.6dC | 783.91±6.61aA | 19.72±0.3bB | 285.36±2.79bB | 7.74±0.6bB | 74.13±2.38bB |
|  | UGJ 4% | 42.55±1.27dB | 4.66±0.04abB | 60.66±1.4dB | 29.07±0.6eC | 664.74±7.4dB | 17.78±0.36dB | 254.16±3.04cB | 6.90±0.28cB | 64.61±2.26dB |
|  | UGJ 7% | 57.65±1.79aC | 5.84±0.04aB | 75.34±1.23aB | 37.44±0.19aC | 775.51±3.52bA | 21.24±0.1aB | 300.98±1.91aB | 9.13±0.32aB | 84.23±2.28aB |
|  | UGJ 8% | 49.21±1.01bB | 4.07±0.03bC | 62.17±1.3cB | 32.10±0.41bB | 621.59±6.38eB | 18.38±0.23cB | 253.03±3.1cB | 7.92±0.32bB | 71.09±2.53cB |
|  | UGJ 12% | 49.92±0.68bB | 3.27±0.02bC | 60.32±0.57dB | 30.82±0.62cC | 593.82±6.43fB | 17.55±0.29dB | 240.06±2.99eB | 7.01±0.78cB | 64.41±3.28dB |
|  | UGJ 16% | 50.43±1.87bB | 3.22±0.02bB | 58.44±1.29eB | 30.00±0.5dC | 544.01±5.72gB | 16.86±0.25eB | 227.94±2.7fB | 7.17±0.2cB | 68.92±1.98cB |
| E-FM | CK | 27.29±0.73gD | 4.75±0.09cB | 46.42±0.73dC | 19.29±0.14gD | 554.27±4.33bD | 7.95±0.05cC | 179.55±1.57dC | 2.54±0.1dC | 31.07±0.94eD |
|  | UGJ 2% | 32.32±0.35eD | 5.58±0.07aB | 51.51±0.42bD | 22.50±0.02eD | 604.76±2.32aD | 14.42±0.26aC | 215.80±1.43bD | 3.07±0.08cD | 38.19±1.08cD |
|  | UGJ 4% | 33.89±0.36dC | 4.44±0.01dB | 49.2±0.39cD | 23.86±0.32cD | 555.09±2.68bD | 8.98±0.34bcD | 190.82±2.31cD | 3.11±0.11bcC | 37.82±1.23cD |
|  | UGJ 7% | 40.57±0.69aD | 5.19±0.03bC | 55.21±0.59aD | 28.08±0.15aD | 606.13±3.36aD | 15.58±0.16aD | 229.15±2.06aD | 3.54±0.09aDE | 44.11±1.17aD |
|  | UGJ 8% | 35.91±0.58bD | 3.53±0.03eE | 46.69±0.45dD | 24.72±0bD | 494.49±2.63cD | 11.66±3.63bC | 190.45±11.9cD | 3.45±0.13aD | 40.71±1.26bD |
|  | UGJ 12% | 29.99±0.23fE | 3.07±0.02fD | 38.87±0.24fE | 21.88±0.21fD | 433.70±3.26eE | 10.34±2.65bcDE | 170.69±6.8eE | 3.02±0.12cD | 34.71±1.25dE |
|  | UGJ 16% | 35.18±0.62cE | 2.99±0gC | 45.39±0.58eD | 23.13±0.29dD | 462.01±3.83dD | 11.12±3.64bC | 180.12±12.12dD | 3.23±0.09bD | 39.66±1.25bE |
| DO | CK | 23.32±2.04dE | 4.61±0.02aBC | 46.56±0.3dC | 14.72±0.1fE | 568.46±3.88dC | 6.60±0.07bD | 179.23±1.25dC | 2.49±0.06cC | 30.96±0.91dD |
|  | UGJ 2% | 28.00±2.14cdE | 4.67±0.22aBC | 56.37±1.04aC | 20.22±1.04eE | 664.51±3.44aC | 12.10±6.94abD | 228.32±23.06bC | 3.99±0.65abC | 45.31±0.92bC |
|  | UGJ 4% | 27.64±7.81cdD | 3.05±0.3cE | 50.74±0.62bC | 21.12±0.23dE | 607.94±6.19cC | 11.68±6.18abCD | 216.88±20.32bcC | 3.22±0.16bcC | 41.22±1.73cC |
|  | UGJ 7% | 31.88±0.24bcE | 3.98±0.02bE | 56.99±0.22aC | 24.77±0.47aE | 647.79±2.55bC | 17.95±0.18aC | 249.61±1.37aC | 5.05±1.84aC | 51.34±4.3aC |
|  | UGJ 8% | 31.88±6.84bcE | 0.71±0fG | 48.95±0.38cC | 22.29±0cE | 548.52±3.85eC | 11.89±5.14abC | 209.20±13.65cC | 3.98±0.81abC | 44.72±2.86bcC |
|  | UGJ 12% | 34.95±0.23bD | 1.72±0.03eE | 45.35±0.3eC | 21.63±0.13dD | 514.98±4.29fC | 11.62±4.73abCD | 199.14±11.72cC | 3.99±0.65abC | 43.72±1.18bcC |
|  | UGJ 16% | 40.11±4.67aD | 1.9±0.01dE | 49.35±0.11cC | 23.34±0.33bD | 518.68±0.92fC | 12.15±3.5abC | 206.61±8.68cC | 5.38±0.73aC | 54.59±1.02aC |
| BW | CK | 21.97±1.53efE | 4.47±0.72aBC | 37.64±0.6dD | 12.36±0.07gF | 442.38±1.16eE | 8.63±2.79bC | 152.48±9.21eD | 1.84±0.07eD | 23.30±0.57dE |
|  | UGJ 2% | 20.04±0.24fF | 4.02±0.01bC | 47.27±2.32aE | 15.67±1.8fF | 566.12±2.49aE | 15.18±0.03aC | 216.17±0.22aD | 2.86±0.07cD | 36.86±0.51bD |
|  | UGJ 4% | 23.25±0.52deE | 3.54±0.34cD | 43.47±0.42cE | 17.07±1.17eF | 499.31±0.98cE | 13.85±0.17aC | 192.71±1.13bD | 2.64±0.09dD | 33.71±0.81cE |
|  | UGJ 7% | 29.04±0.05bF | 4.69±0.02aD | 46.78±0.26abF | 19.14±0.22dF | 502.80±0.36bE | 14.53±0.42aE | 195.45±0.26bE | 2.86±0.38cE | 36.35±0.71bE |
|  | UGJ 8% | 31.57±0.13aE | 3.65±0.01bcD | 42.96±0.28cE | 20.80±0.01bF | 430.14±2.21gE | 8.04±0.03bD | 158.08±1.1eE | 3.03±0.08cE | 33.95±1.01cE |
|  | UGJ 12% | 25.09±0.32cF | 0.71±0eF | 43.06±0.13cD | 19.72±0cE | 451.22±0.67dD | 13.37±0.04aC | 184.52±0.04cD | 3.34±0.03bD | 36.99±0.5bD |
|  | UGJ 16% | 33.45±4.59aE | 2.06±0.01dD | 45.65±0.14bD | 22.11±0aE | 433.05±1.28fE | 10.45±4.07bC | 170.33±11.69dD | 3.66±0.15aD | 42.48±1.21aD |
| AG-1 | CK | 3.37±0.52gF | 0.56±0dE | 4.77±0.7dF | 2.09±0.36fG | 57.83±9.64fG | 1.74±0.33fE | 19.67±3.28fF | 0.33±0.04gF | 3.20±0.55fG |
|  | UGJ 2% | 7.34±0.09cG | 0.64±0.09cdD | 11.57±0.4aG | 6.49±0.08aG | 126.82±0.65aG | 4.57±0.03aE | 47.93±0.19aF | 0.94±0cE | 8.19±0.12cG |
|  | UGJ 4% | 7.01±0.01eF | 0.68±0.14cdF | 8.75±0.93bG | 5.14±0.1cG | 98.10±0.27cG | 3.48±0.03cE | 36.34±0.18cF | 0.74±0.02dE | 6.31±0.05dH |
|  | UGJ 7% | 7.18±0.15dG | 0.76±0.05bcF | 9.02±0.01bG | 4.54±0.11dG | 87.05±1.6dG | 2.92±0.04dG | 32.31±0.56dG | 0.65±0.02eF | 6.14±0.11dF |
|  | UGJ 8% | 5.85±0.03fF | 0.81±0.08bF | 6.37±0cG | 3.24±0eG | 61.10±0.14eG | 2.05±0.02eE | 22.62±0.09eG | 0.48±0.01fG | 4.53±0.06eG |
|  | UGJ 12% | 8.02±0.02bG | 0.67±0.14cdF | 11.20±0.59aG | 6.49±0.03aF | 107.60±0.52bG | 3.96±0.09bF | 41.95±0.07bG | 1.05±0bF | 8.66±0.09bG |
|  | UGJ 16% | 9.41±0.05aF | 0.98±0.11aF | 11.34±0.32aF | 6.07±0.03bF | 95.73±0.06cG | 3.54±0.04cD | 36.57±0.04cF | 1.09±0.01aE | 9.10±0.06aG |
| AG-2 | CK | 1.59±0.18fG | 0.33±0.01cE | 2.35±0.12fG | 1.04±0.05eH | 28.25±0.05fH | 0.63±0.19eE | 9.31±0.14fG | 0.92±0.02bE | 40.85±1.24aC |
|  | UGJ 2% | 4.13±0.25bcH | 0.42±0.04bD | 5.64±0.06aH | 2.52±0.02bH | 60.74±0.12aH | 1.88±0.01aE | 22.21±0.08aG | 0.73±0.01dE | 30.86±0.59cE |
|  | UGJ 4% | 3.38±0.08dG | 0.40±0.01bG | 4.07±0.11eH | 1.99±0.03dH | 42.93±0.32dH | 1.28±0.02cdE | 15.01±0.21dG | 0.71±0.03dE | 28.92±1.35dF |
|  | UGJ 7% | 3.88±0.14cH | 0.43±0.01bG | 4.75±0.07cH | 2.27±0.02cH | 47.22±0.35cH | 1.41±0.03cH | 17.03±0.18cH | 1.10±0.02aF | 37.71±0.76bE |
|  | UGJ 8% | 2.22±0.05eG | 0.35±0cH | 2.17±0gH | 1.11±0.01fH | 22.82±0.23gH | 0.42±0.04fE | 7.19±0.14gH | 0.81±0.02cG | 39.73±1.49aD |
|  | UGJ 12% | 4.54±0.26aH | 0.42±0.02bG | 5.49±0.06bH | 2.78±0.01aG | 52.96±0.34bH | 1.65±0.01bF | 18.61±0.13bH | 0.46±0.01eG | 17.66±0.37fF |
|  | UGJ 16% | 4.22±0.02bG | 0.48±0.02aH | 4.55±0.10dG | 2.26±0.02cG | 40.07±0.17eH | 1.18±0.05dD | 12.85±0.17eG | 0.47±0.01eF | 20.83±0.72eF |

a. The different small letters indicate a significant difference (*p*<0.05) among different groups. b. The different capital letters indicate a significant difference (*p*<0.05) among different stages. c. “ND” represents “Not Detected”; “UGJ” represents “unripe grape juice”; “Dp” represents “Delphinidin-3-O-glucoside”; “Cy” represents “Cyanidin-3-O-glucoside”; “Pt” represents “Petunidin-3-O-glucoside”; “Pn” represents “Peonidin-3-O-glucoside”; “Mv” represents “Malvidin-3-O-glucoside”; “Pn-acet” represents “Peonidin-3-O-(6-acetyl)-glucoside”; “Mv-acet” represents “Malvidin-3-O-(6-acetyl)-glucoside”; “tPn-coum” represents “Peonidin-3-O-(trans-6-coumaryl)-glucoside”; “tMv-coum” represents “Malvidin-3-O-(trans-6-coumaryl)-glucoside”.
